# Priming self-assembly pathways by stacking block copolymers

**DOI:** 10.1038/s41467-022-34729-0

**Published:** 2022-11-14

**Authors:** Sebastian T. Russell, Suwon Bae, Ashwanth Subramanian, Nikhil Tiwale, Gregory Doerk, Chang-Yong Nam, Masafumi Fukuto, Kevin G. Yager

**Affiliations:** 1 Center for Functional Nanomaterials, Brookhaven National Laboratory, Upton, NY 11973 USA; 2 Department of Materials Science and Chemical Engineering, Stony Brook University, Stony Brook, NY 11794 USA; 3 National Synchrotron Light Source II, Brookhaven National Laboratory, Upton, NY 11973 USA

**Keywords:** Molecular self-assembly, Self-assembly, Polymers

## Abstract

Block copolymers spontaneously self-assemble into well-defined nanoscale morphologies. Yet equilibrium assembly gives rise to a limited set of structures. Non-equilibrium strategies can, in principle, expand diversity by exploiting self-assembly’s responsive nature. In this vein, we developed a pathway priming strategy combining control of thin film initial configurations and ordering history. We sequentially coat distinct materials to form prescribed initial states, and use thermal annealing to evolve these manifestly non-equilibrium states through the assembly landscape, traversing normally inaccessible transient structures. We explore the enormous associated hyperspace, spanning processing (annealing temperature and time), material (composition and molecular weight), and layering (thickness and order) dimensions. We demonstrate a library of exotic non-native morphologies, including vertically-oriented perforated lamellae, aqueduct structures (vertical lamellar walls with substrate-pinned perforations), parapets (crenellated lamellae), and networks of crisscrossing lamellae. This enhanced structural control can be used to modify functional properties, including accessing regimes that surpass their equilibrium analogs.

## Abstract (web summary)

Self-assembly of block-copolymers yields nanoscale structures in a facile way, but the diversity of structures is limited. Here, the authors demonstrate how block copolymer layering can be used to access new non-equilibrium morphologies.

## Introduction

Self-assembly is a robust paradigm for the preparation of nanoscale patterns^1–3^, where molecules spontaneously organize into well-defined structures in order to minimize energy. Block copolymers (BCPs) are a well-studied example, where chemically-distinct blocks along the polymer chain drive towards phase separation, but the covalent link between the blocks frustrates this. The energetic compromise is to form a nanoscale morphology dictated by the chain architecture. Because BCPs are amenable to simple solution processing and thermal annealing, these materials are in principle scalable to manufacturing contexts where nanostructured coatings are rapidly applied over macroscopic areas^4–6^. Self-assembly is intrinsically driven by energy-minimization, which tends to emphasize simple structures with minimal surface area and maximal symmetry^7^. As a result, equilibrium assembly affords only a limited selection of structural motifs such as spheres, cylinders, and lamellae^8,9^. Non-equilibrium self-assembly has been employed to organize materials through reaction-diffusion systems^10–14^, active matter assemblies^15–17^, or biochemical networks^18–20^. For BCP materials, a variety of directed self-assembly methods have been developed to reduce the defect density or prepare new structures, including epitaxial alignment^21,22^, responsive layering^23,24^, layer-by-layer stacking^3,25,26^, solvent-vapor annealing^27–29^, and external fields^30–36^. Recently, manipulating processing histories has emerged as a potential way to exploit non-equilibrium phenomena through pathway-dependent self-assembly^37–42^. While promising, this concept faces many challenges. Control over a system’s entire processing history is arduous and, for a given processing history, there is no guarantee that pathway-dependence will lead to novel structural motifs, making it is unclear how to capitalize on this non-equilibrium aspect.

Here, we demonstrate a new method to control non-equilibrium self-assembly—engineering the system’s initial configuration. Rather than starting from a disordered and homogeneous as-cast state, we prepare a non-trivial initial configuration from which we initiate self-assembly. This effectively primes self-assembly, selecting a particular pathway for subsequent ordering. Starting in structurally disparate initial configurations—dramatically different locations along the overall material state-space periphery—allows access to regions in the self-assembly landscape that are otherwise inaccessible. Here, we demonstrate the formation of initial states based on layering of distinct block copolymer materials into stacked films, using sequential blade-coating. Subsequent thermal annealing of these bilayers of course intermixes the materials. However, because phase separation and self-assembly occur alongside the interdiffusion process, a variety of new transient structural motifs appear. This approach is versatile in that it can combine BCP intrinsic information (molecular architecture) with extrinsic processing control (material stratification, layer order, and mixture composition). Accessing new regions of the state-space not only leads to structural novelty, but also to constructs that possess enhanced material properties compared to their corresponding equilibrium structure. These results underline that pathway-priming with only a small set of starting materials can produce a surprisingly large variety of non-native structures.

## Results

### Pathway priming BCP self-assembly method

**Fig. 1:**
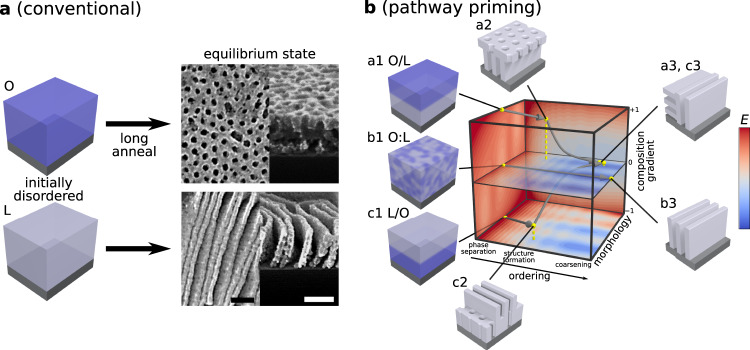
Traditional self-assembly vs. pathway priming. **a** In traditional BCP thin-film processing, disordered, homogenous films cast from solution are annealed for long times at high temperatures to achieve conventional equilibrium morphologies (e.g., cylinders or lamellae). **b** Non-trivial layered initial configurations (a1 and c1) are used to initiate self-assembly pathways that pass through non-equilibrium transient states (a2 and c2) and progress towards final morphologies after long annealing times (a3 and c3). These pathways are distinct from the corresponding non-layered blend (b1 to b3). Scale bars are 100 nm.

Conventional BCP self-assembly (Fig. 1a), is focused on reaching the equilibrium morphology, using thermal or solvent annealing to provide the system with the mobility necessary for ordering to proceed. One typically starts the self-assembly process from a trivial initial configuration—the unordered, homogenous state that arises naturally from film casting. Ordering proceeds through initial local phase separation throughout the material, followed by organization of these phases into local patterns of well-defined symmetry and repeat-spacing, followed by progressive coarsening of the structure through defect annihilation which increases grain size. The non-equilibrium morphologies that appear in such an ordering history are essentially highly defective versions of the equilibrium state, which are of limited interest^38,42^. However, one can instead start the self-assembly from a prescribed initial configuration. While the outcome after long annealing is expected to be the equilibrium state associated with that chain composition, one might observe intermediate states with non-native structural motifs. To study this concept, we generated layered initial film configurations, where distinct BCP materials are stacked on top of one another (processing scheme in Supplementary Fig. 1). We identified regimes of blade-coating processing conditions (solvent, concentration, flow velocity) where one can cast a polymer film on top of an existing film with minimal dissolution of the first layer or interdiffusion of the two layers (details in Supplementary Fig. 2). This method can thus generate arbitrary BCP stacks as initial configurations and can control the degree of initial layer interdiffusion. While film transfer methods^43–46^ can also be used to create layered states, blade coating is a simple means of controlling layer thicknesses (${h}_{1},{h}_{2}$) and thus overall BCP mixture composition ($\phi$).

This protocol was exploited to investigate the non-equilibrium self-assembly associated with pathway-primed polystyrene-*block*-poly(methyl methacrylate) diblock copolymer (PS-*b*-PMMA) combinations. For simplicity, we limit the initial configuration scope to $\le 2$ BCP materials (L = Lamellae, C = Cylinders, O = inverse cylinders), $\le 2$ layers (single-layer and bilayers), and use a neutral substrate coating (roughly equal interaction strength with PS and PMMA) that promotes vertical orientation of the morphology. We adopt the nomenclature MXX for BCP materials, where M indicates the preferred phase and XX the molecular weight (in kg mol^–1^). Each initial configuration leads to a different self-assembly pathway through the multidimensional material state-space, and thus a distinct set of transient states (Fig. 1b). Depending on the priming (i.e., the point at the state-space frontier where self-assembly is initiated), energy barriers can direct self-assembly into kinetically trapped states (Fig. 1b, pathway a & c), or a purely downhill energy pathway can allow self-assembly to progress towards the equilibrium morphology (Fig. 1b, pathway b). In principle, one can stop the assembling mixture at any point along the pathway and thereby select a particular transient state, simply by quenching below the glass transition temperature (~100 °C for PS-*b*-PMMA). These quenched transient states were converted into inorganic replicas using infiltration synthesis, which allows them to be easily imaged using scanning electron microscopy (SEM)^47^. Thus, one can take advantage of the enormous non-equilibrium material state-space to search for exotic non-native structures.

### Transforming generic materials into exotic morphologies

Layered assemblies of BCP materials were found to evolve into non-native morphologies during thermal annealing. Three illustrative examples are shown in Fig. 2, where we provide experimental realization and structural confirmation using coarse-grained molecular dynamics (MD) simulations (Supplementary Fig. 3). Through exploration of a wide variety of preparation and annealing conditions, we discovered a host of structures both stable and transient (Fig. 3 and Supplementary Figs. 4–24) and identified a set of unifying design principles (Table 1). These non-equilibrium ordering considerations act collectively to determine the structures that arise, though the relative contribution of each principle varies depending on the particular pathway.

**Fig. 2:**
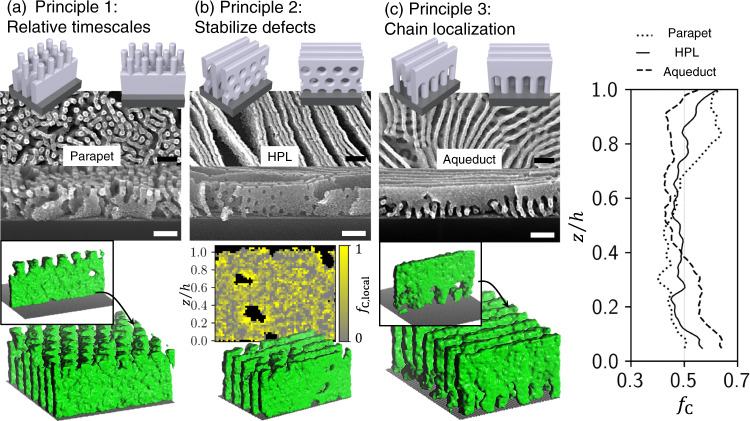
Non-native structures. Three examples of non-native structures that appear as a result of pathway-primed self-assembly. For each, top-view and cross-sectional SEM are shown (scale bars 100 nm) on top, and corresponding molecular dynamics (MD) simulations below. **a** Local phase separation can occur on a faster timescale than chain interdiffusion, giving rise to layered configurations such as parapet, formed by annealing a C/L bilayer (L75 bottom layer was preannealed 1200 s at 250 °C, second layer C67, bilayer annealed 15 s at 250 °C). **b** The existence of two chain types allows otherwise defective structures to be stabilized, such as perforations in an HPL (L36/C67 bilayer annealed 300 s at 270 °C). A map of the local chain composition (*f*_C,local_) highlights how C chains concentrate around high-curvature perforations, stabilizing them. **c** Interfacial energetics can drive towards non-uniform distribution of chains, which leads to the formation of the aqueduct morphology (bilayer where each layer is a blend of C67:L75, annealed 1200 s at 250 °C). (right) The fraction of C chains (*f*_C_) through the film depth (normalized height *z*/*h*) highlights how the different morphologies have distinct underlying chain spatial distributions.

**Fig. 3:**
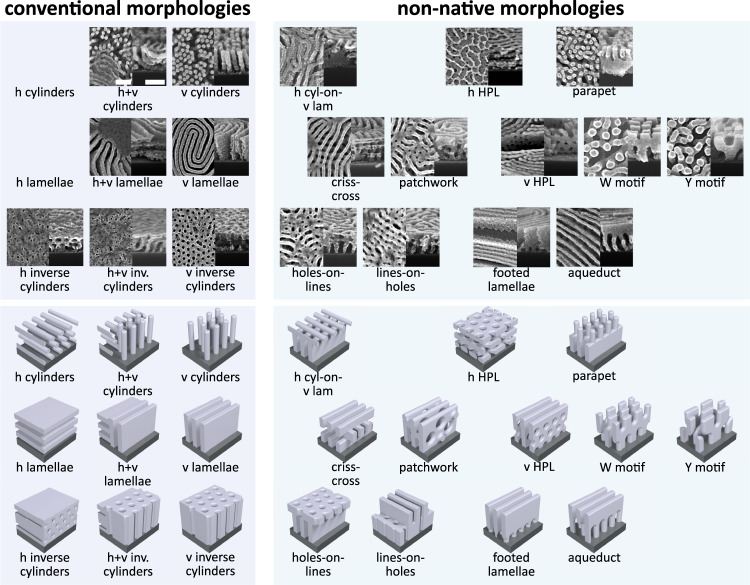
Catalog of nanostructure diversity. A set of 19 different structures including conventional (left) and non-native (right) motifs found using pathway priming self-assembly. For each state, a top-down and cross-sectional scanning electron microscopy image (top) and a schematic representation (bottom) are shown. Example conditions specifying how each pattern can be achieved are provided in Supplementary Figs. 5–24. Scale bars are 100 nm and apply to all images.

**Table 1:** Pathway priming structural design principles

1	At early annealing times, local microphase segregation occurs before complete film-wide chain intermixing.
2	Structural defects (e.g., perforations, domain anti-alignment, or mixed motifs) are stabilized by blending; i.e., because chains can reorganize during BCP layer intermixing.
3	Preferential segregation of one chain type at interfaces can induce structurally asymmetric morphologies.
4	Large energy barriers in the self-assembly landscape can kinetically trap some pathways.

Upon thermal annealing, BCP chains undergo microphase segregation, a rapid and spontaneous phase separation process that occurs before complete layer intermixing. Indeed, the phase segregated domains hinder chain diffusion^48^. Therefore, at early annealing times, layered BCP films exhibit morphologies that can be thought of as a stacking of the individual equilibrium phases (Table 1, principle 1). A key example of this behavior can be seen for early annealing times of C/L bilayers, which form a parapet-like structure with vertical cylinder-like protrusions connected to underlying vertical lamellae sheets (Fig. 2a and Supplementary Fig. 15). This can be understood as the two layers (cylinder-forming chains on top, and lamellae-forming chains on bottom) each locally forming their preferred equilibrium structure. This arises since local phase separation is faster than chain diffusion over the scale of the film thickness. However, one also notices that these two structures are registered and connected, a hallmark of the energy-minimization of self-assembly. MD simulations confirm the formation of parapet structures for C/L bilayers at early annealing times, and provide a means of quantifying the chain distribution as a function of film depth (*z/h*). The parapet structure is associated with a concentration gradient, wherein the top of the film is C-rich and the bottom is L-rich (Fig. 2, right). It is interesting to note how the parapet structure persists even after substantial chain interdiffusion, since there is a substantial energy barrier for converting a preexisting morphology. However, as will be discussed later, the parapet structure is ultimately a transient (kinetic) structure. Principle 1 also explains the formation of other transient structures, such as holes-on-lines and lines-on-holes (Fig. 3 and Supplementary Figs 21 and 22).

As annealing proceeds, the layers intermix, and transient structures evolve into new kinds of morphologies. The mixing of distinct chain types allows the system to stabilize morphological defects by redistributing the constituent chains (Table 1, principle 2). For instance, cylinder chains can localize in high-curvature defects in a lamellae phase, stabilizing perforations in the lamellar sheets. This can give rise to the formation of a vertically-oriented hexagonally perforated lamellae (vHPL) morphology (Fig. 2b and Supplementary Fig. 18). MD simulations can track the distribution of individual chains, confirming that the cylinder chains tend to organize around the perforations (c.f. the relative fraction of C chains, *f*_C,local_, Fig. 2b). More generally, the coexistence of two chain types provides self-assembly with greater configurational freedom. The distinct chain types can redistribute spatially to minimize energy of a broader range of structural motifs, giving rise to a correspondingly larger range of morphologies. Defect stabilization plays a role in the formation of lamellar walls with substrate-pinned perforations (footed lamellae, Supplementary Fig. 23), the crisscross motif (Supplementary Fig. 16), and the poorly-ordered patchwork motif (Supplementary Figs. 17 and 27). For example, the O/C and C/O systems form highly disordered patchwork states (Supplementary Figs. 17, 27a–b) at early annealing times, likely due to stabilization of high-curvature defects arising from the combination of C and O chains, which accommodate curvature of opposite sign.

**Fig. 4:**
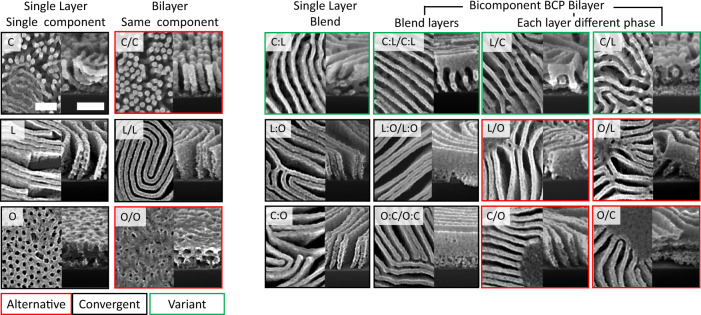
BCP state-space complexity impacts pathway convergence. SEM images (top-down and cross-section) of same-component and bi-component BCP bilayers compared to their single-layer analogs (annealed at 250 °C for 1200 s). Different evolution behaviors can be observed (denoted by color of outline box): some pathways converge together into a common final morphology (black), while other pathways lead to variants of a particular motif (green). Some self-assembly pathways become kinetically trapped in metastable states (red). Scale bars are 100 nm.

At long annealing times, the differences between constituent chain types (especially the lengths of the minority and majority blocks) can result in preferential chain segregation, leading to enhanced concentration of a particular chain type at film interfaces^49^ (Table 1, principle 3). This phenomenon can have a major impact on the BCP morphology, stabilizing asymmetric structures such as the aqueduct morphology, which can be thought of as lamellae sheet merged with high-curvature cylinder-like structures at the substrate interface (Fig. 2c and Supplementary Fig. 24). MD simulations confirm the formation of this structure, and enable identification of the underlying chain ratio through the film depth (*f*_C_, Fig. 2 right). In particular, the aqueduct morphology is associated with an excess of C chains near the substrate interface (and a corresponding depletion in the upper parts of the film). The aqueduct morphology is most easily accessed by priming a L/C bilayer, which of course prescribes C chains in the lower half of the film. However, we also observe formation of aqueduct morphologies at long annealing times for blends of C and L, and even for C/L bilayers (Fig. 4 and Supplementary Fig. 4). This suggests that the observed asymmetric chain concentration is stable and thermodynamically preferred. The spontaneous and persistent concentration gradient can be rationalized based on the entropic preference for small chain-ends to localize at interfaces, combined with the slight enthalpic preference of C chains for the substrate coating used in this work (see methods section). Chain concentration gradients also explain the formation of the W and Y morphologies (Supplementary Figs. 19 and 20), which can be thought of as a lamellar mid-layer connecting cylinder-rich regions forming at the substrate and air interfaces.

Self-assembly is generically prone to kinetic trapping; in pathway-priming, many evolution histories become bounded by kinetic barriers in the material state-space (Table 1, principle 4). Contrived initial configurations afforded by priming, combined with the defect-tolerance afforded by blending, exacerbate kinetic trapping. For example, when layering films formed from different BCP types, we observed the frequent formation of mixed alignments, such as coexistence of vertical and horizonal lamellae (Supplementary Fig. 8). Many of the observed structures (Fig. 3) are non-equilibrium; either transient states that ultimately convert into some other structure, or metastable states that persist only due to the large energetic barrier to reorganization of the film’s entire morphology. These pathway-dependent aspects are discussed in further detail in a later section.

### Preparation history influences structure formation

Exploring the full set of bilayered films formed by C, L, and O highlights the diversity of transient structures and the sensitivity to preparation conditions (Supplementary Figs. 4, 25, and 26). We now describe some preparation histories and their impact on structure formation. An interesting case to consider are bilayers formed by the same material stacked on top of itself (Fig. 4, second column). One might assume that bilayers of the same material would yield identical evolution to a single thick film of that material (Fig. 4, first column). Instead, we observe key differences. For instance, a film (160 nm) of C material yields a cylinder phase with random cylinder orientations; whereas a C/C bilayer (each layer 80 nm) instead yields a well-defined vertical morphology across the entire film thickness. The O/O bilayer forms a horizontal inverse cylinder phase, while the O single-layer film forms a vertical orientation. These differences can be ascribed to the weak and latent discontinuity in BCP chain distribution at the film midplane, which is evidently sufficient to drive independent ordering in the top and bottom of the film for a short time^38^ (principle 1). The two layers must then merge as annealing proceeds. In the case of C/C, for instance, each layer initially orients vertically (as typically observed in very thin films) and then these two aligned morphologies register and merge into a single vertical phase through the entire film thickness. In the case of L/L bilayers, the two layers naturally anti-align^50–52^, forming a crisscross structure (Supplementary Fig. 16).

As previously discussed, creating bilayers from disparate materials yields a host of more complex morphologies; most notably the formation of parapet structures from C/L bilayers, and the formation of the aqueduct morphology from many preparation conditions (L/C, C:L blend, and long-time annealing of C/L). It is noteworthy that inverted layering do not yield the same structure (at early annealing times) despite having the same overall chain composition (Supplementary Fig. 4). One can expand the range of possible structures by layering BCPs of different molecular weight, and thus dissimilar repeat spacings. For bilayers of longer C chains and shorter L chains (${N}_{{{{{{\rm{CYL}}}}}}}/{N}_{{{{{{\rm{LAM}}}}}}}\ge 1$, where *N*_CYL_ and *N*_LAM_ are the cylinder and lamellar BCP degree of polymerization respectively), aqueduct is the dominant structure (Supplementary Figs. 29a–c, e–h). In this molecular weight regime, increasing ${\phi }_{{{{{{\rm{CYL}}}}}}}$ deepens the aqueduct arches near the substrate interface, consistent with the larger C fraction. In the regime where C chains are shorter than L chains (${N}_{{{{{{\rm{CYL}}}}}}}/{N}_{{{{{{\rm{LAM}}}}}}}\, < \,1$), varying ${\phi }_{{{{{{\rm{CYL}}}}}}}$ similarly influences the extent of cylinder-like high-curvature regions. However, a different suite of non-native structures arise—W and Y-shaped motifs (Supplementary Figs. 19, 20, and 29j–m). These can be thought of as a combination of the parapet and aqueduct morphologies, where a central lamellar region connects to perforated lamellae at the top and bottom of the film. This structure forms because C chains localize to both interfaces (principle 3) and stabilize perforations in the vicinity (principle 2). Molecular weight disparity provides an additional control over non-native structure formation, and access to compositionally asymmetric morphologies.

Annealing temperature provides another parameter for controlling structure formation. As an example, L36/C67 bilayers annealed at lower temperatures (*T* < 250 $^\circ {{{{{\rm{C}}}}}}$) yield the expected aqueduct morphology (Supplementary Figs. 29b–c and 30a, b). At higher temperatures (*T* ~ 270 $^\circ {{{{{\rm{C}}}}}}$) this bilayer forms hexagonally perforated lamellae (HPL); either vertically-oriented (*T* ~ 270 $^\circ {{{{{\rm{C}}}}}}$, Supplementary Fig. 31) or horizontally oriented (*T* > 280 $^\circ {{{{{\rm{C}}}}}}$, Supplementary Fig. 30d). The vertical HPL structure (Supplementary Fig. 18) is a previously unreported morphology that arises only through the delicate stabilization afforded by intermixed C and L chains (principle 2). The temperature-dependence in this regime likely arises from the temperature-dependence of chain segregation (principle 3). Temperature provides a means of adjusting the relative strength of these effects; at higher temperature the entropic driving force becomes stronger, and thus L enrichment at both the top and bottom interfaces dominates, giving rise to the vHPL morphology where the C-rich perforations are driven to the center of the film.

Even more contrived initial configurations can be realized by pre-annealing one or both layers, generating initial morphological ordering that governs subsequent co-ordering during bilayer annealing. For instance, the most well-ordered version of the parapet morphology is produced by annealing an initial L layer to yield vertical lamellae, then adding a disordered C layer, and then annealing this bilayer for a short time (5 s at 250 °C; Fig. 2a and Supplementary Fig. 15). The top layer C material phase-separates to form vertical cylinders, which must immediately align and register with the underlying lamellar lines (principle 1). Subsequent interdiffusion of chains helps to stabilize these connections (principle 2). We also used film transfer methods to construct bilayers where both top and bottom were annealed and pre-ordered, leading to transient structures consistent with principle 1. For instance, stacking a layer of vertical cylinders on top of a layer of vertical lamellae essentially constructs a parapet-like structure that persists for short annealing times (<5 s). However, the strong preexisting order in the top layer conflicts with that in the bottom, resulting in the transient formation of horizontal cylinders on top of vertical lamellae (~15 s annealing, Supplementary Fig. 13) as the system evolves towards the ultimate aqueduct morphology.

### Kinetic phenomena associated with multidimensional assembly landscape

Pathway priming leverages non-trivial starting configurations to navigate the material state-space. At early annealing times this leads to pathway-dependent behavior and, as annealing proceeds, self-assembly pathways can either converge to a common long-time anneal motif or become kinetically trapped in a metastable structure (Fig. 4). Thus, priming encodes for unique transient-states and in some cases allows one to circumvent or exploit energy barriers in the material state-space (principle 4). As previously noted, bilayers formed by stacking the same material yield a different set of structural states than the single layer of that material (Fig. 4, first two columns). This is a manifestly kinetic (pathway-dependent) effect, since the overall chain composition—and even the chain makeup through the film depth—are identical. Similarly, different structures are sometimes observed between layered and blended systems with the same overall chain composition (Fig. 4, right panel).

**Fig. 5:**
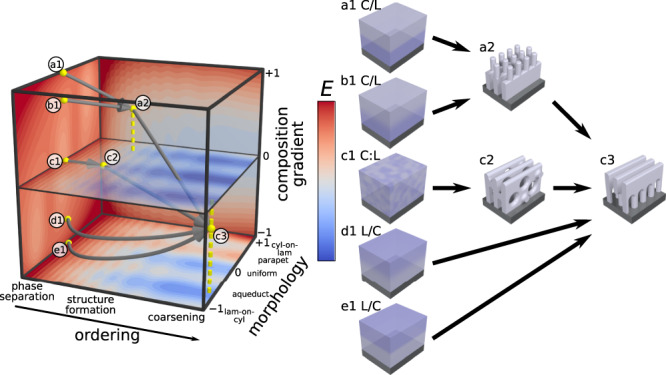
Material state-space complexity. Proposed explanation for the effect of primed states on self-assembly pathway diversity. The shape of the graph should be regarded as schematic only; nevertheless, it is based on the underlying self-assembly phenomena. Pathway primed self-assembly proceeds along three general axes: composition gradient ($\lambda$), morphology ($\eta$), and ordering ($t$). The pathway primed initial configurations (*t* = 0; states a1–e1) capture the different layer orders and width of interface ($\lambda$ = 0 represents a blend film). Some systems are principally convergent (such as combinations of C and L), with different initial states passing through transient states but ultimately reaching a common final state. In other systems, kinetic barriers may separate ordering histories.

This kinetic trapping can be contrasted against sets of pathways that instead converge to a common endpoint. For instance, both the L single-layer and the L/L bilayer initial configurations form vertical lamellae after long annealing times. The various combinations of C and L material all eventually form structural variants of the aqueduct morphology (Fig. 4, green borders), though they exhibit distinct transient states (Supplementary Fig. 32a) along their respective pathways. The contrasting behaviors (kinetic trapping vs. convergence) highlight the complexity of assembly landscapes. Priming can either initiate self-assembly pathways into regions bounded by kinetic barriers which will be trapped in metastable states (Fig. 4, red), or into regions featuring a converging downhill landscape flowing into a common long-time anneal morphology (Fig. 4, black). Bilayers made by stacking different BCP types broadens the accessible material state-space, in comparison to pure component films, by increasing the dimensionality of the assembly landscape (Fig. 5). Layer order allows for constructing initial configurations that are farther apart in the state-space (e.g., C/L, C:L blend, L/C) enabling a clear distinction between self-assembly pathway.

A material state-space is the structural manifestation of the associated non-equilibrium self-assembly energy landscape, which is dependent on physical, chemical, and processing parameters. The assembly landscape is a very high-dimensional construct that must capture all the degrees-of-freedom associated with phase separation, chain intermixing, and morphological structuring as a function of depth within the film. Nevertheless, a simplified understanding can be provided by collapsing this space into a three-dimensional assembly landscape (Fig. 1b and Fig. 5) with axes: ordering history ($t$), morphology ($\eta$), and composition gradient ($\lambda$) along the film normal. Ordering history captures structural evolution, which includes rapid phase separation and the formation of local structural motifs, followed by rearrangement to form a morphology, followed by reordering and finally coarsening of ordered grains as defects are eliminated. Layering is captured by the composition gradient axis, which quantifies the asymmetry in the underlying chain distribution (the blend is $\lambda=0$ by definition). Finally, the morphology axis captures the emergent nanoscale ordering; for instance, we can define *η* = 0 to be uniform morphology through the film depth, and use the extremes of this axis to capture morphologies that appear distinct near the top vs. bottom of the film. In this schematic, primed BCP initial configurations initiate self-assembly on the $t=0$ plane (Fig. 5a1–e1), where the blend starts at the origin ($t=0$, $\eta=0$, and $\lambda=0$) and the bilayers start at a nonzero $\lambda$ values (i.e., $\lambda \, > \, 0$ for C/L and $\lambda \, < \,0$ for L/C). At the early stages of self-assembly, microphase segregation occurs before complete chain intermixing, leading to transient state morphologies with nonzero $\lambda$ and $\eta$ values ($\eta \, > \, 0$ for C/L and $\eta \, < \, 0$ for L/C [Fig. 5a2, c2]). As self-assembly proceeds, diffusion will cause the layers to intermix, driving all self-assembly pathways toward the $\lambda=0$ plane. Figure 5 demonstrates a convergent state-space where all initial configurations tend towards the same energy minimum and thus the same long-time anneal morphology (Fig. 5c3). In contrast, Fig. 1b represents a material state-space containing regions bounded by kinetic barriers. This process is schematically represented through three separate initial configurations forming diverse early annealing time structures that progress into two different long-time anneal structures (Figs. 1, a3 & c3 vs. b3).

**Fig. 6:**
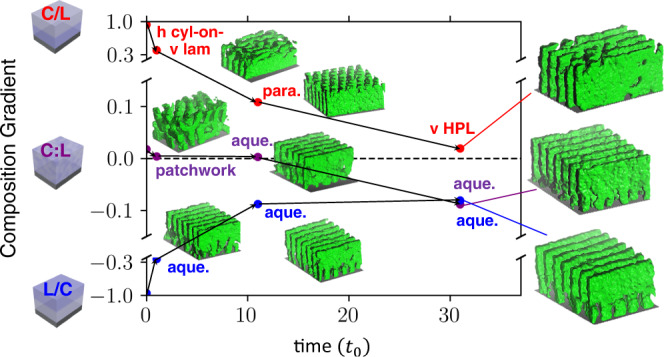
Kinetic trapping effects. MD simulation ordering histories for C/L, C:L, and L/C. The structural evolution is denoted by the change in BCP chain composition through the film depth, as a function of simulation time (in units of ${t}_{0}=0.06\times {10}^{6}\tau$, which is the approximate timescale at which morphologies first become discernable). The influence of kinetic barriers is demonstrated by the eventual convergence of the L/C and C:L systems, versus the kinetic trapping of the C/L configuration.

Transient states can be interrogated using molecular dynamics (Fig. 6). When comparing structural evolution for the C:L blend and the C/L and L/C bilayers, the pathways are clearly distinct. Most obviously, early structures are quite different, and reflect the initial layering (principle 1). All three pathways seem to be evolving towards the aqueduct morphology (consistent with experiments); yet the rate of progress is notably different. Within the studied simulation time, the C:L blend and the L/C bilayer rapidly reach the aqueduct state, with a stable enrichment of C chains near the substrate ($\lambda=-0.1$). However, the C/L bilayer reached a (defective) vHPL morphology within the simulated time window, and was evolving only slowly thereafter. These slow kinetics can be ascribed to the succession of energy barriers associated with breaking apart or migrating stable perforations at the top or middle of the film, in order to reform the substrate-pinned channels associated with the aqueduct morphology. The morphology and underlying chain distribution are intimately related; the simulated C/L bilayer exhibits a lingering enrichment of C in the top of the film ($\lambda \, > \, 0$, c.f. Fig. 2 right). These results confirm that priming a system farther away from the stable end-state (for that chain composition) leads to a more tortuous ordering pathway; this correspondingly provides an opportunity to identify and isolate novel transient states.

### Pathway-dependent material properties

**Fig. 7:**
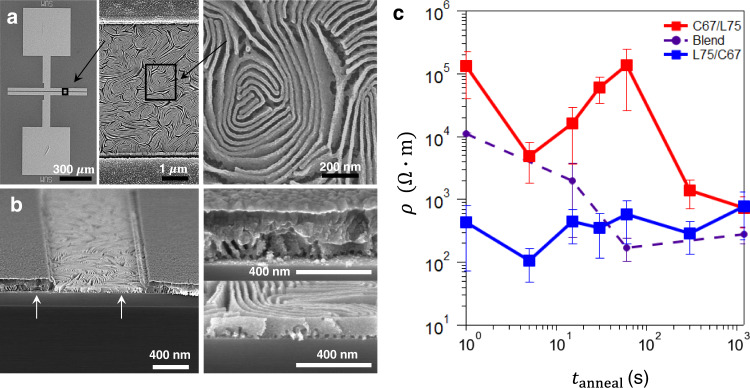
Electrical properties as a function of structure. **a** Top-down SEM and **b** SEM cross-sections of electrical devices fabricated on the L75/C67 bilayer configuration annealed for ${t}_{{{{{{\rm{anneal}}}}}}}=1200$ s at 250 $^\circ {{{{{\rm{C}}}}}}$. **c** The electrical resistivity of metal oxide nanostructures fabricated from BCP bilayers are compared to those from the blend (error bars are standard deviation from multiple replica samples). Material layering, as well as BCP annealing, greatly influence the morphology, and thereby the resultant electrical connectivity after conversion into an inorganic replica.

Pathway-dependent assembly has a direct impact on functional properties. Using C67 and L75 chains, three separate initial configurations (C/L, C:L, L/C) with identical ${\phi }_{{{{{{\rm{C}}}}}}67}$ and total film thickness were prepared to investigate the impact of structure on electrical transport properties. Transient structures were extracted at different annealing times and converted to aluminium-doped ZnO (AZO) semiconductor replicas (see methods section for details), to measure the corresponding in-plane electrical resistivity ($\rho$). Electrical devices were fabricated by depositing patterned metal contacts on top of the AZO replicas with excellent structural preservation (Fig. 7a, b; full structure characterization provided in Supplementary Figs. 33–35).

In the long annealing time regime (${t}_{{{{{{\rm{anneal}}}}}}}=1200\,{{{{{\rm{s}}}}}}$), the observed $\rho$ values are nearly identical and independent of initial configurations, which is expected due to the convergent assembly of the three systems into an aqueduct morphology. However, the structural diversity along different assembly pathways manifests in a strong $\rho$ process history dependence. At the early stages of self-assembly (${t}_{{{{{{\rm{anneal}}}}}}}=5$ s), the blend and C/L configurations have similar electrical resistivities, which are almost two orders of magnitude larger than the L/C configuration. Electrical conductivity is of course directly tied to the internal connectivity of the underlying morphology, which is influenced by both morphology (some motifs provide better percolation pathways), orientation (in-plane vs. out-of-plane), and structural order (to provide long pathways for conduction). Using a sequential etching protocol, we visualized the orientation and connectivity as a function of depth (see methods). The C/L and blend configurations form domains predominantly oriented vertically, leading to poor in-plane conductivity. In contrast, domains formed by L/C bilayers are predominantly horizontal, resulting in the measured improvement to the in-plane conductivity. In general, mesh-like networks formed from in-plane interconnected domains should exhibit excellent conductivity owing to in-plane paths and connections between layers that allow current flow around high-resistance regions^25,47,53,54^. Priming the system in the blend or C/L configuration starts the self-assembly pathway farther from the aqueduct motif, thus assembly traverses transient states with a larger structural diversity during annealing. This leads to correspondingly large variations in $\rho$. In particular, the C/L bilayer initially converts from poorly-ordered initial phase-separated state (high resistivity at 1 s) into a parapet morphology whose structural order provides conduction pathways (low resistivity at 5 s). Subsequent reordering into the aqueduct state disrupts order and transits through intermediate states that exhibit mixed three-dimensional collections of cylinders and lamellae (poor long-range connectivity leading to a maximum in resistivity at 60 s). By contrast, the L/C bilayer orders directly into the aqueduct morphology, leading to a relatively constant trend in $\rho$. Annealing the L/C configuration for longer times eliminates transient domain interconnectivity while increasing the structural correlation length translating to the observed $\rho$ process history dependence in the extracted AZO replicas. The L/C configuration represents a self-assembly pathway that contains transient states with enhanced functional properties in comparison to the long-time anneal morphology.

## Discussion

Priming self-assembly takes advantage of the structural diversity in material state-spaces to naturally access non-native morphologies. This was done by engineering a non-trivial initial configuration and thereby starting self-assembly pathways in different locations of the material state-space. Using this strategy, a catalog of structurally diverse morphologies was identified by assembling a handful of commercially available BCP materials. The observed structural diversity can be rationalized from a small set of design principles, which explain the appearance transient states (parapet, holes-on-lines, lines-on-holes, and patchwork) and metastable morphologies (aqueduct, vertical perforated lamellae, and W and Y-shaped motifs). Priming self-assembly pathways provide a means of identifying kinetic barriers in assembly landscapes, and circumventing them using contrived initial configurations. It was demonstrated that processing history can have a dramatic impact on functional properties. Careful control of surface nanostructure and material type opens the door to applications in high-performance coatings with tunable electrical^25^, optical^55,56^, and wettability^57,58^ properties. Using only a limited supply of starting materials, priming self-assembly pathways shows potential to afford libraries of non-equilibrium motifs possessing non-native structural characteristics and enhanced functional properties.

## Methods

### Substrates

To control the domain orientation relative to the substrate and air interfaces, silicon substrate surfaces were neutralized by applying a PS-*r*-PMMA-OH random copolymer (provided by the Dow Chemical Company). The brush, after being functionalized to the Si surface, creates a chemically neutral surface (similar interaction energy with PS and PMMA)^59–61^. The brush composition was 61 mol% styrene, determined using ^13^C NMR, with total molecular weight M_n_ = 9.2 kg mol^–1^ and polydispersity M_w_/M_n_ = 1.35 (determined by gel permeation chromatography relative to PS standards)^23^. The neutral brush (1% wt. in PGMEA) was applied by spin coating at 1500 rpm, followed by annealing at 250 °C for 5 min to promote surface grafting, and finally rinsing in toluene to remove excess material.

### Materials

Block copolymer (Polymer Source, Inc.) films were applied to the functionalized Si wafers via blade coating at fixed blade velocity. PS-*b*-PMMA block copolymers were selected as model materials, since this class of materials is well-studied and tend to exhibit ordering similar to other coil-coil BCPs. The PS-*b*-PMMA solutions varied from 1 to 3% wt. in toluene depending on the desired film thickness. The blade was a glass microscope slide (25 × 75 mm^2^) at a fixed angle (8°) with respect to the sample stage and positioned ∼50 µm above the sample surface. Each experiment used $30\,{{\upmu }}{{{{{\rm{L}}}}}}$ of sample solution and blade velocities ranged from $15-35\,{{{{{\rm{mm}}}}}}\; {{{{{\rm{s}}}}}}$^−^^1^ to yield films of a uniform thicknesses. The film thicknesses studied in this work span a wide range of between ∼$75-200$ nm. All film thicknesses were determined using ellipsometry (J.A. Woollam Co., M-2000). The block copolymer materials used in these experiments are described in Table 2. Propylene glycol methyl ether acetate (PGMEA), acetone, toluene, trimethylaluminum (TMA), and diethylzinc (DEZ) were purchased from Sigma-Aldrich and used as received. Film annealing was carried out using a variable temperature nitrogen-purged hotplate. Replicate samples were processed and compared to confirm that structure formation is reproducible for a given layering and annealing history.

**Table 2:** Relevant material properties for commercial BCPs

Polymer	${{MW}}_{{PS}}$ [kg/mol]	${{MW}}_{{PMMA}}$ [kg/mol]	${N}_{{PS}}$	${N}_{{PMMA}}$	${R}_{g,{PS}}$ [nm]	${R}_{g,{PMMA}}$ [nm]	$D$
C67	46	21	443	210	15.5	8.9	1.09
L75	38	37	366	368	14.0	11.7	1.06
O71	20	51	193	510	10.2	13.8	1.07
L36	18	18	173	180	9.6	8.2	1.07
L211	105	106	1010	1060	23.3	19.9	1.13

The dispersity index ($D$) and block molecular weights (${{MW}}_{i}$) were provided by polymer source and determined using gel permeation chromatography and H^1^ NMR analysis, respectively. The radii of gyrations were approximated as gaussian coils using ${R}_{g,i}={b}_{i}\sqrt{{N}_{i}/6}$, where ${b}_{i}$ and ${N}_{i}$ are the Kuhn length and degree of polymerization of block $i$, respectively.

### SEM

Thin film surface morphology was characterized using a Hitachi S-4800 Scanning Electron Microscope (SEM) with 10 kV accelerating voltage.

### Image analysis

We computationally analyzed the self-assembled patterns from SEM images using the Python programming language and exploiting libraries for image manipulation (Python Image Library), numerical computations (numpy^62^), and plotting (matplotlib^63^). To evaluate the relative fractional areal coverage of lamellar versus cylindrical morphologies for blended block copolymer thin films exhibiting both phases, we used image thresholding and particle-counting to isolate image structures, identifying lines (lamellae) and dots (cylinders) using a size cutoff. These characteristic structures were grown using a flood-fill method to segment the entire image into regions expressing the two morphologies. The resulting image was then overlaid atop the original to isolate one of the two morphologies for further analysis. We determined the average domain spacing by applying a Fast Fourier Transform (FFT) to the image and fitting the fundamental peak position in the one-dimensional, circularly averaged spectrum. The average grain size for vertical lamellar morphologies (line patterns) was estimated using established methods^64^.

### Metal oxide replication of BCP nanostructures

The infiltration synthesis of ZnO and AlO_x_ into self-assembled BCP thin film templates was carried out in a commercial ALD system (Cambridge Nanotech, Savannah S100) at 85 °C using diethyl zinc (DEZ) and trimethyl aluminum (TMA) as respective metal-organic precursors along with water as an oxidant. The microdose infiltration synthesis (MDIS) cycle of AlO_x_ was conducted as follows. The precursor exposure period was 5 min but the precursor (both TMA and water) dosing pulse was repeated every 30 s during the exposure period (total 10 repeated microdosing pulses). The pulse sequencing was determined using a strategy described elsewhere^47^. The complete AlO_x_ infiltration synthesis protocol followed 6 ZnO MDIS cycles to ensure the structural integrity of PMMA inorganic replica extending down to the Si substrate. The ZnO MDIS followed the same infiltration protocol using DEZ as the precursor and water as an oxidant. Prior to the ZnO infiltration synthesis, a single AlO_x_ MDIS cycle was performed to seed the growth of ZnO in the PMMA domains. The ZnO MDIS infiltration synthesis was followed by the initial removal of the organic polymer matrix by oxygen plasma ashing (20 W; 100 mTorr; 5 min; room temperature) and the further consolidation of the inorganic matrix and the removal of carbon impurities by O_2_ RTP treatment at 600 °C for 5 min (Modular Process Technology, RTP-600S).

### Electrode fabrication and device measurements

Silicon substrates with 300 nm thermal oxide were used for electrical characterization. The metal electrodes were patterned on top of the AZO replica using lift-off protocol described elsewhere^65^. Two-probe I–V characteristics of the devices were measured using an electrical probe station (Signatone) equipped with a dark box and a high-precision semiconductor parameter analyzer (Agilent). The electrical resistances (*R*) were extracted from the I–V curves via a linear fit (ohm’s law) and the electrical resistivity $(\rho )$ was determined using $\rho\,=R \times A/l$, where A is the sample cross-sectional area (determined using the product of electrode length (500 ${{\upmu }}{{{{{\rm{m}}}}}}$) and film thickness) and $l$ is the spacing between electrodes.

### Molecular dynamics simulations

We prepared molecular dynamics (MD) models for cylindrical and lamellar diblock copolymer chains and used them to construct films of different configurations. Individual block copolymer chains are described by a coarse-grained bead-spring model^66^, and every chain is composed of two different blocks, A and B, resulting in a A-*b*-B BCP chain. Each block is a series of beads of the corresponding type, and the two different blocks are covalently bonded. The substrate was constructed of a single layer of hexagonally packed beads (type S). The interactions between beads are governed by bonded (FENE—finite extensible nonlinear elastic) and non-bonded (12-6 LJ – Lennard Jones) potentials. We set the self-cohesion energy between A blocks to be slightly larger than that between B blocks (which yields a system where the minority phase has a slightly larger surface tension than the majority phase), and the cohesion energy between A and B blocks was set to be smaller than between like beads (which yields BCP phase separation). The S beads mimic the neutral experimental substrates by using non-bonded interactions with BCP beads, the strengths of which were determined by the experimental composition ratio of the random copolymer brush^67,68^. The initial, disordered film configuration of every film was generated by using a self-avoiding random walk algorithm. We use standard reduced units: $\sigma$ for distance, $m$ for mass, $\epsilon$ for energy, $\tau$ for time where $\tau={\sigma (m/\epsilon )}^{1/2}$. The dimensions of the simulation box are $57\sigma \times 90\sigma \times 80\sigma$. The height of $80\sigma$ was chosen to be much larger than the film thickness ($\approx 47\sigma$) to allow the formation of a polymer-air interface. We used the LAMMPS simulation package made available by Sandia National Laboratory^69^. Simulations were carried out using the NVT ensemble and a timestep of $0.006\tau$ with periodic boundary conditions imposed along $x$ and $y$ axes. The film was equilibrated at $T=1.2\epsilon /{k}_{B}$, where ${k}_{B}$ is the Boltzmann constant. Molecular snapshots were captured using the VMD software package^70^. Details about film configurations and potentials are available in the Supplementary Information.

## Supplementary information

Supplementary Information

## Data Availability

Primary data are available within the Article and Supplementary Information. Source data for manuscript graphs is provided as Supplementary Information. The complete set of original Scanning Electron Microscope image files are available through the Material Data Facility repository at: 10.18126/hx77-iub1. All data are also available by request to the corresponding author. Source data are provided with this paper.

## Source data

Source Data
