# Supplementary material for: Priming self-assembly pathways by stacking block copolymers
Source: Nat Commun. 2022 Nov 14;13:6947. doi: 10.1038/s41467-022-34729-0 (PMC9663688; doi:10.1038/s41467-022-34729-0)
Supplement: Supplementary file 1 — Supplementary Information [file 41467_2022_34729_MOESM1_ESM.pdf]

Supplementary Information for:

Priming Self-Assembly Pathways by Stacking Block Copolymers

Sebastian T. Russell[1], Suwon Bae[1], Ashwanth Subramanian[2], Nikhil Tiwale[1], Greg Doerk[1], Masafumi Fukuto[3], Chang-Yong Nam[1,2], Kevin G. Yager[*1]*

[1]Center for Functional Nanomaterials, Brookhaven National Laboratory, Upton, New York 11973, United States
[2]Department of Materials Science and Chemical Engineering, Stony Brook University, Stony Brook, New York 11794, USA
[3]National Synchrotron Light Source II, Brookhaven National Laboratory, Upton, NY 11973
[*]kyager@bnl.gov

Supplementary Methods

Solution processing and imaging of BCP bilayer films

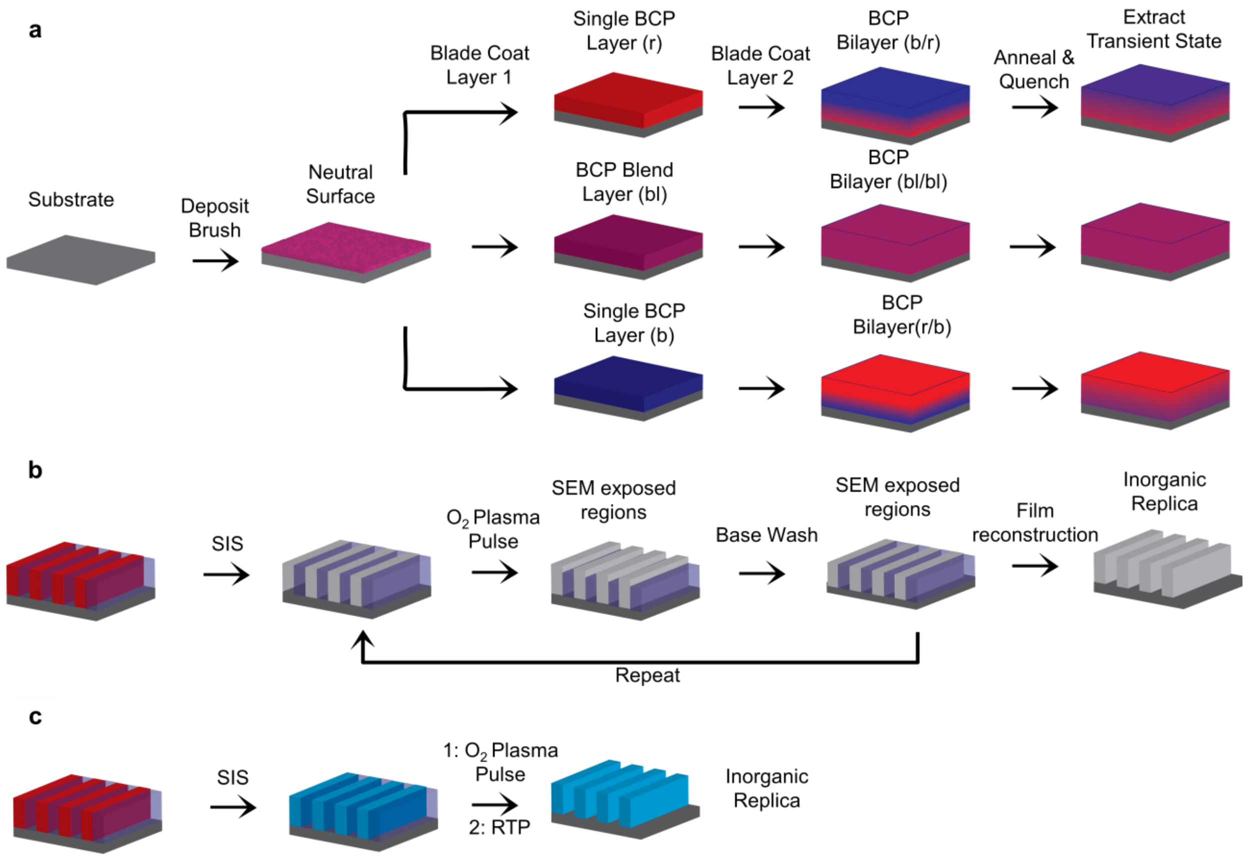

Figure 1| (a) BCP bilayers are formed by first functionalizing a neutral brush onto the Si substrate. Then, two BCP layers are blade coated sequentially where the second layer is coated directly on top of the previous layer creating a stratified bilayer stack. The BCP bilayers are then annealed and quenched after prescribed annealing times to extract kinetically trapped morphologies. The thickness of layer 1 and layer 2 were determined using ellipsometry and film compositions are determined via layer fraction. (b) An inorganic replica of a given structure can be generated through sequential infiltration synthesis (SIS), followed by brief exposure to O_2 plasma which partially etches the BCP film. To image the sub-surface structure, films are progressively etched using an aqueous basic solution to expose the successive depths of the film. (c) The entire BCP replica can be imaged (top-down and cross-section) using a tailored SIS protocol that seeds the growth of zinc oxide with alumna oxide (AZ0)[1]. The protocol is provided in the experimental section.

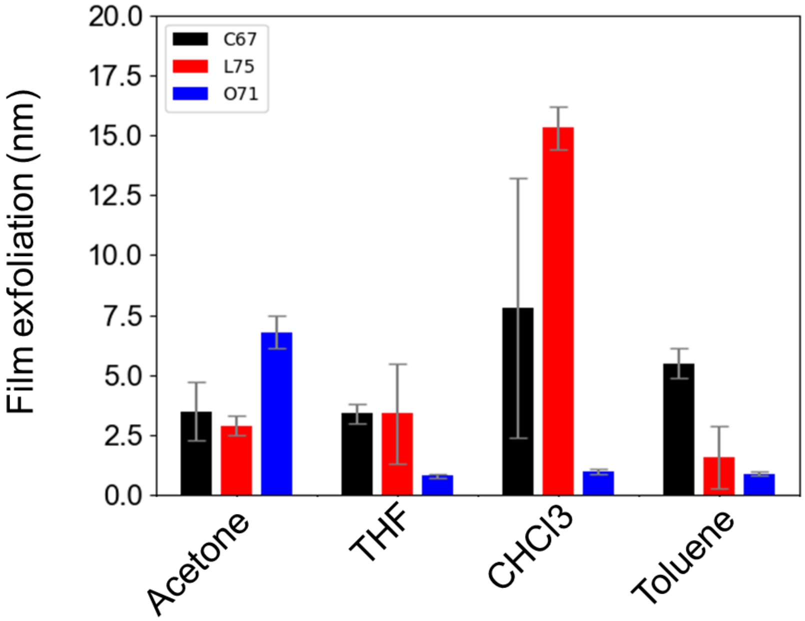

Figure 2| The film exfoliation is the estimated amount of BCP material (C67, L75, or O71) removed during the bilayer coating process. For sequential coatings, the first BCP film was prepared through blade coating and the film thickness was measured ($\mathrm{h_{intial}}$). Then, instead of coating a second layer of material, pure solvent was drawn over the polymer layer using the same blade velocity (30 mm/s). The BCP layer thickness was measured again ($\mathrm{h_{final}}$). The film exfoliation was calculated as $\mathrm{h_{intial}} - \mathrm{h_{final}}$. This process was repeated using different organic solvents and each BCP examined in this study. Toluene was chosen as the coating solvent for this study as it showed minimal film exfoliation across all BCP of interest and evaporates quickly resulting in minimal disturbance to the underlying layer. This measurement should only be considered as an estimate of film removal during bilayer coating, since the specific coating conditions (polymer concentration, solvent evaporation rate, etc.) may influence dissolution of the initial layer. For instance, a concentrated polymer solution will tend to induce less film swelling and less film dissolution than a pure solvent.

Molecular dynamics simulation

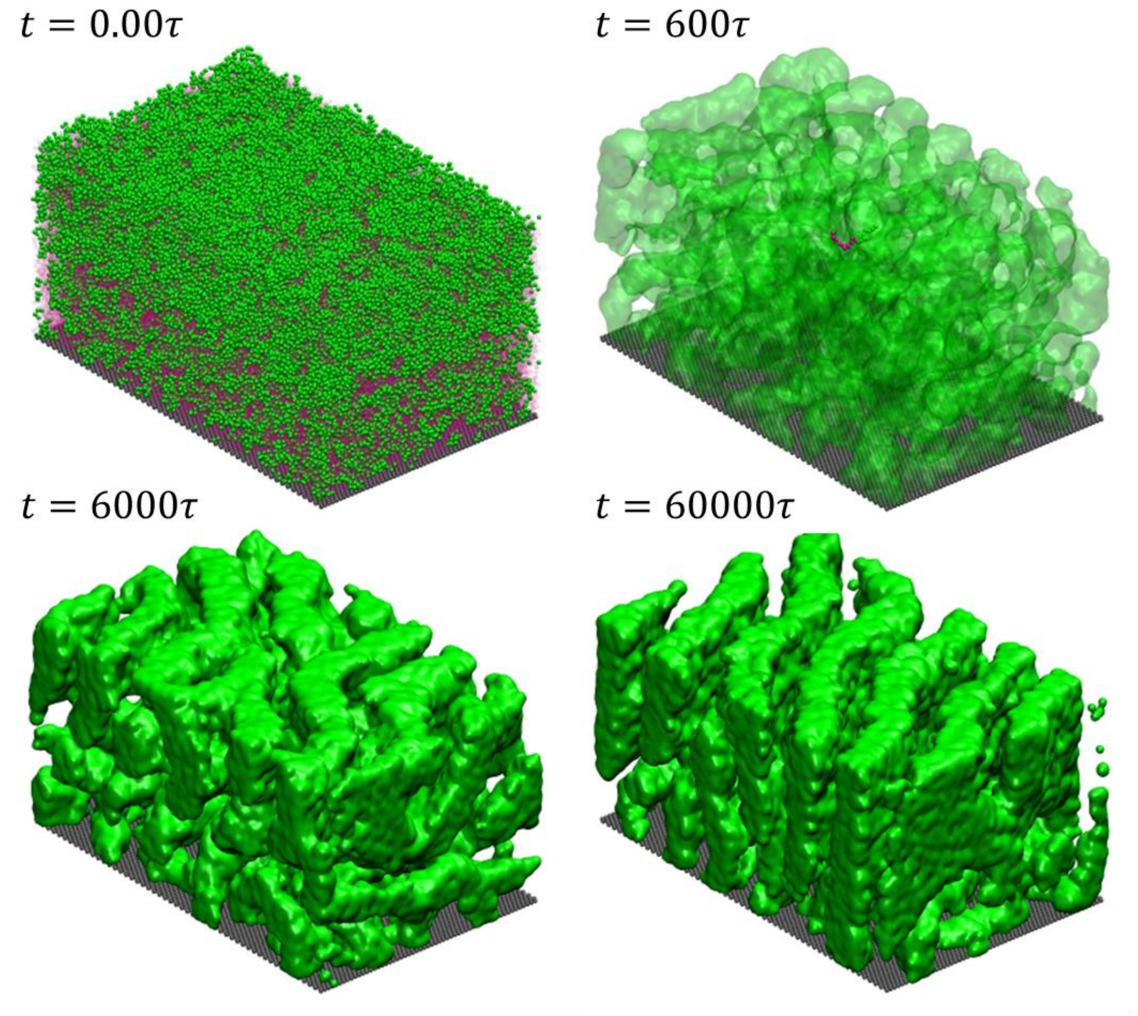

Figure 3| Example of coarse-grained molecular dynamics (MD) structural evolution. An L/C film evolves from the initial disordered state into a well-define morphology. The initial configuration was rendered using beads to highlight the scale of individual BCP chains (minority A blocks colored green, majority B blocks magenta and transparent). The morphology at $t = 600\tau$ was rendered transparent, and a single BCP chain is drawn to highlight the relative scales. The late-time renders are based on an isosurface of the minority phase (A block) material (this visualization scheme is used in the main text)

Coarse grained molecular dynamics (MD) BCP films were prepared using 20-bead-long C chains and/or 22-bead-long L chains (unless specified otherwise). Every bead has unit mass ($1m$).

	Phase 1			Phase 2			
	N	f_A	n	N	f_A	n	ϕ_1
C/C	20	0.25	4800	20	0.25	4800	0.5
L/L	22	0.5	4364	22	0.5	4364	0.5
C/L, L/C, and C:L	20	0.25	4800	22	0.5	4364	0.5
C/L(longer)	20	0.25	4800	30	0.5	3200	0.5

Table 1| The configuration of each bilayer or blended film where N is the number of beads per chain, f_A is the fraction of block A – the chain architecture, n is the number of chains, and ϕ_1 is the mass fraction of phase 1.

The bonded interactions are governed by the finite extensible nonlinear elastic (FENE) potential, which is described by an expression in terms of r, the distance between adjacent beads, and 5 parameters as written in Equation S1. k is a spring constant, R_0 is a maximum length, ϵ_{ij} is the strength of interaction between a bead of type i and a bead of type j, σ_{ij} is the finite distance at which the inter-bead potential is zero, and r_c is the cutoff radius.

$$U_{\mathrm{FENE}}(r) = -0.5kR_0{}^2 \ln\left(1 - \frac{r^2}{R_0{}^2}\right) + 4\epsilon_{ij}\left[\left(\frac{\sigma_{ij}}{r}\right)^{12} - \left(\frac{\sigma_{ij}}{r}\right)^{6} + \frac{1}{4}\right] \qquad \text{(S1)}$$

where the first and second terms are responsible for attractive and repulsive interactions between covalently bonded beads, respectively. The second term is cutoff and shifted to zero energy at r_c of $\sqrt[6]{2}\sigma$, where it shows its minimum, resulting in repulsive interactions alone.

The non-bonded interactions are dictated by the 12-6 Lennard Jones (LJ) potential, given as a function of r, the distance between a pair of beads, and 3 parameters as written in Equation S2. ϵ_{ij} is the strength of interaction between a bead of type i and a bead of type j, σ_{ij} is the finite distance at which the inter-bead potential is zero, and r_c is the cutoff radius.

$$U_{\mathrm{LJ}}(r) = \begin{cases} 4\epsilon_{ij}\left[\left(\frac{\sigma_{ij}}{r}\right)^{12} - \left(\frac{\sigma_{ij}}{r}\right)^{6}\right], & r \le r_c \\ 0, & r > r_c \end{cases} \quad \text{(S2)}$$

where r_c is the cutoff radius. r_c for the non-bonded interactions was set to $2.5\sigma_{ij}$, which is responsible for the attractive interactions between a pair of beads if the distance is larger than to $2^{1/6}\sigma_{ij}$.

**FENE potential**			
k		$30\epsilon/\sigma^2$	
R_0		1.5σ	
r_c		$\sqrt[6]{2}\sigma_{ij}$	
**LJ potential**			
$\sigma_{\mathrm{AA}} = \sigma_{\mathrm{BB}} = \sigma_{\mathrm{AB}} = \sigma_{\mathrm{SA}} = \sigma_{\mathrm{SB}}$		1.0σ	
r_c		$2.5\sigma_{ij}$	
ϵ_{ij}	A	B	S
A	1.01ϵ	0.5ϵ	$0.7040\epsilon \sim 0.7295\epsilon$
B	0.5ϵ	0.99ϵ	$0.7695\epsilon \sim 0.7940\epsilon$

Table 2| Parameters for FENE and LJ potentials that describe the interactions between beads. σ_{ij} and ϵ_{ij} in the FENE potential share the same values with the LJ potential.

Supplementary Notes

Transient states along the BCP self-assembly pathways

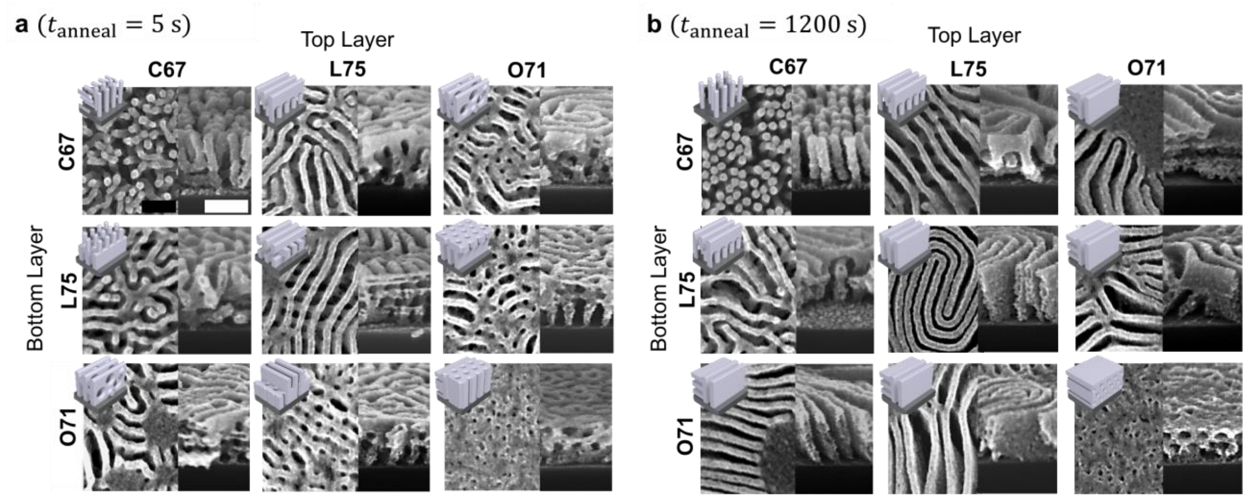

Figure 4| Structural diversity driven by layering different BCP phases. The pathway-primed bilayer configuration is dictated by initial layering, indicated by the column (top layer) and row (bottom layer) intersection. (a) Short annealing times (5 s at 250°C) show that different primed states encode for unique non-equilibrium transient morphologies (top-down and cross-section SEM shown for each). (b) Long annealing times (1200 s at 250°C) show that some initially distinct pathways converge to similar final states. The scale bar (100 nm) applies to all images.

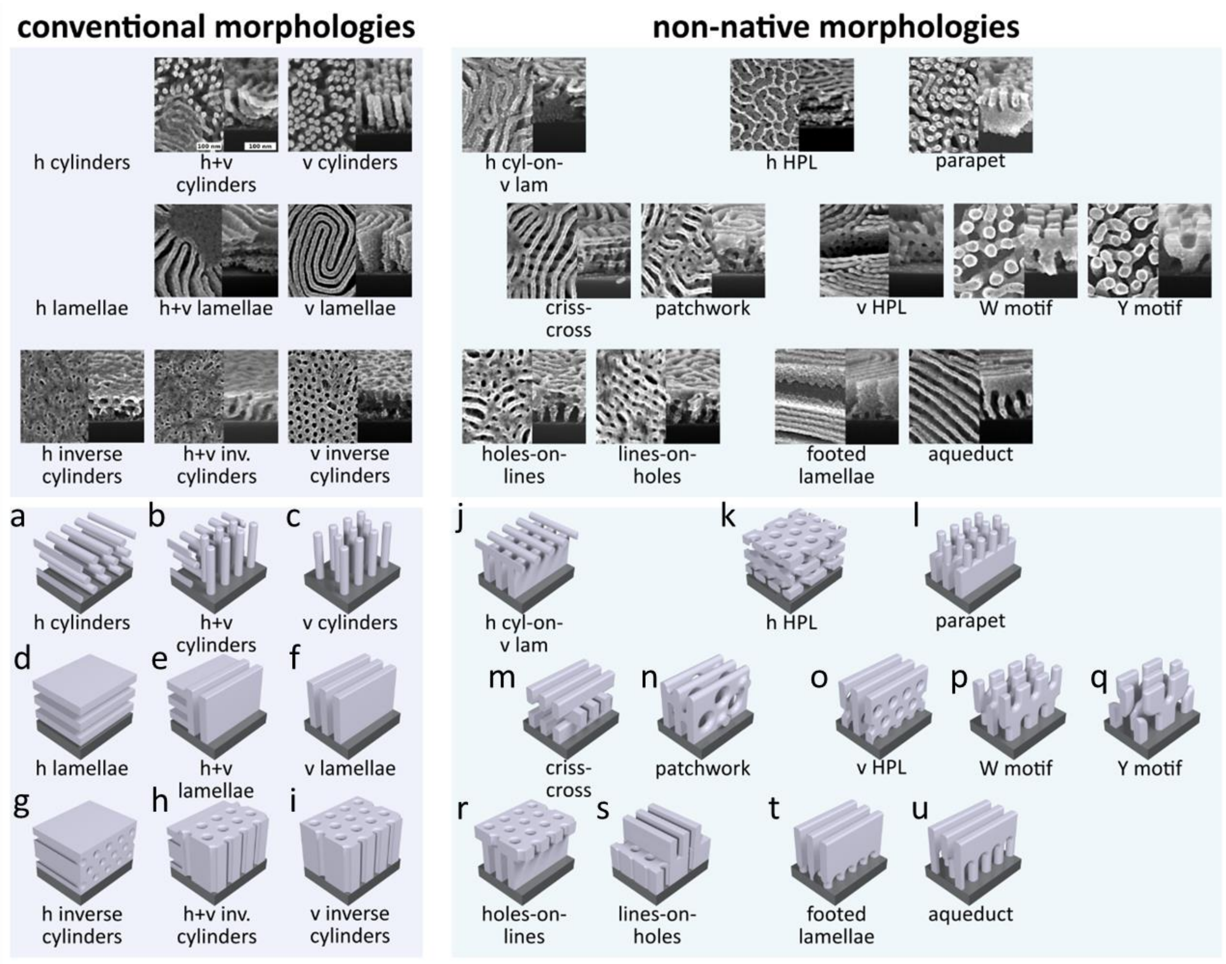

Figure 5| The following is a consolidated list of example conditions that generate each morphology in the diversity catalog. Unless specified otherwise, all conditions assume $\mathrm{T} = 250\ ℃$, $\phi_{\mathrm{CYL}} = 0.5$, C = C67, L = L75, and O = O71 BCPs. (a) N/A; (b) single-layer C, $t_{\mathrm{anneal}} = 1200\ \mathrm{s}$; (c) C/C bilayer, $t_{\mathrm{anneal}} = 1200\ \mathrm{s}$; (d) N/A; (e) L/O bilayer, $t_{\mathrm{anneal}} = 1200\ s$; (f) single-layer L, $t_{\mathrm{anneal}} = 1200\ \mathrm{s}$; (g) bilayer O, $t_{\mathrm{anneal}} = 1200\ \mathrm{s}$; (h) O/O bilayer, $t_{\mathrm{anneal}} = 5\ s$; (i) single-layer O, $t_{\mathrm{anneal}} = 1200\ \mathrm{s}$; (j) vertical C/ vertical L, $t_{\mathrm{anneal}} = 15\ s$; (k) L36/C67 bilayer, $t_{\mathrm{anneal}} = 300\ \mathrm{s}$, $\phi_{\mathrm{CYL}} = 0.68,$ $\mathrm{T}_{\mathrm{anneal}} = 280\ \mathrm{C}$; (l) disordered C/ vertical L, $t_{\mathrm{anneal}} = 15\ \mathrm{s}$; (m) L/L bilayer, $t_{\mathrm{anneal}} = 5\ \mathrm{s}$; (n) C/O bilayer, $t_{\mathrm{anneal}} = 5\ s$; (o) L36/C67 bilayer, $t_{\mathrm{anneal}} = 300\ \mathrm{s}$, $\phi_{\mathrm{CYL}} = 0.64,$ $T_{\mathrm{anneal}} = 280\ \mathrm{C}$; (p) L211/C67 bilayer, $t_{\mathrm{anneal}} = 300\ \mathrm{s}$, $\phi_{\mathrm{CYL}} = 0.35$; (q) L211/C67 bilayer, $t_{\mathrm{anneal}} = 300\ \mathrm{s}$, $\phi_{\mathrm{CYL}} = 0.46$; (r) O/L bilayer, $t_{\mathrm{anneal}} = 5\ s$; (s) L/O bilayer, $t_{\mathrm{anneal}} = 5\ \mathrm{s}$; (t) L/C bilayer, $t_{\mathrm{anneal}} = 1200\ \mathrm{s}$, $\phi_{\mathrm{CYL}} = 0.33$; (u) L/C bilayer, $t_{\mathrm{anneal}} = 300\ \mathrm{s}$.

Structure b: v + h cylinders

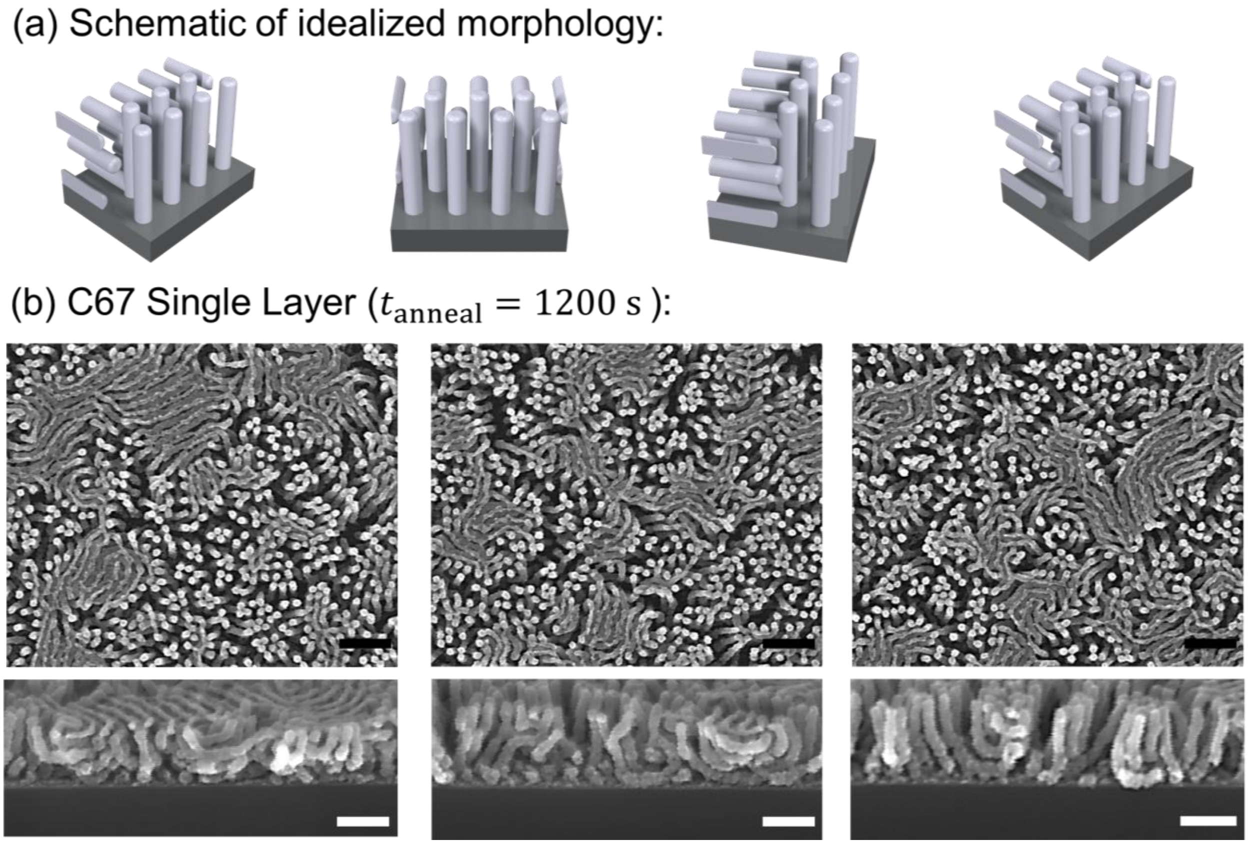

Figure 6| (a) Schematic representation of the mixed vertical and horizontal cylinder morphology. We have provided rendering from multiple angles to be compared with the experimental observations. (b) Top down and perspective SEM images at multiple locations of a bilayer sample (C67 Single Layer, $t_{\text{anneal}} = 1200\ s$, $\mathrm{T} = 250\ ℃$) forming the v + h cylindrical state. We provide multiple locations across the sample surface to highlight that this structural motif persists across the entire samples surface. All scale bars are 100 nm.

Structure c: v cylinders

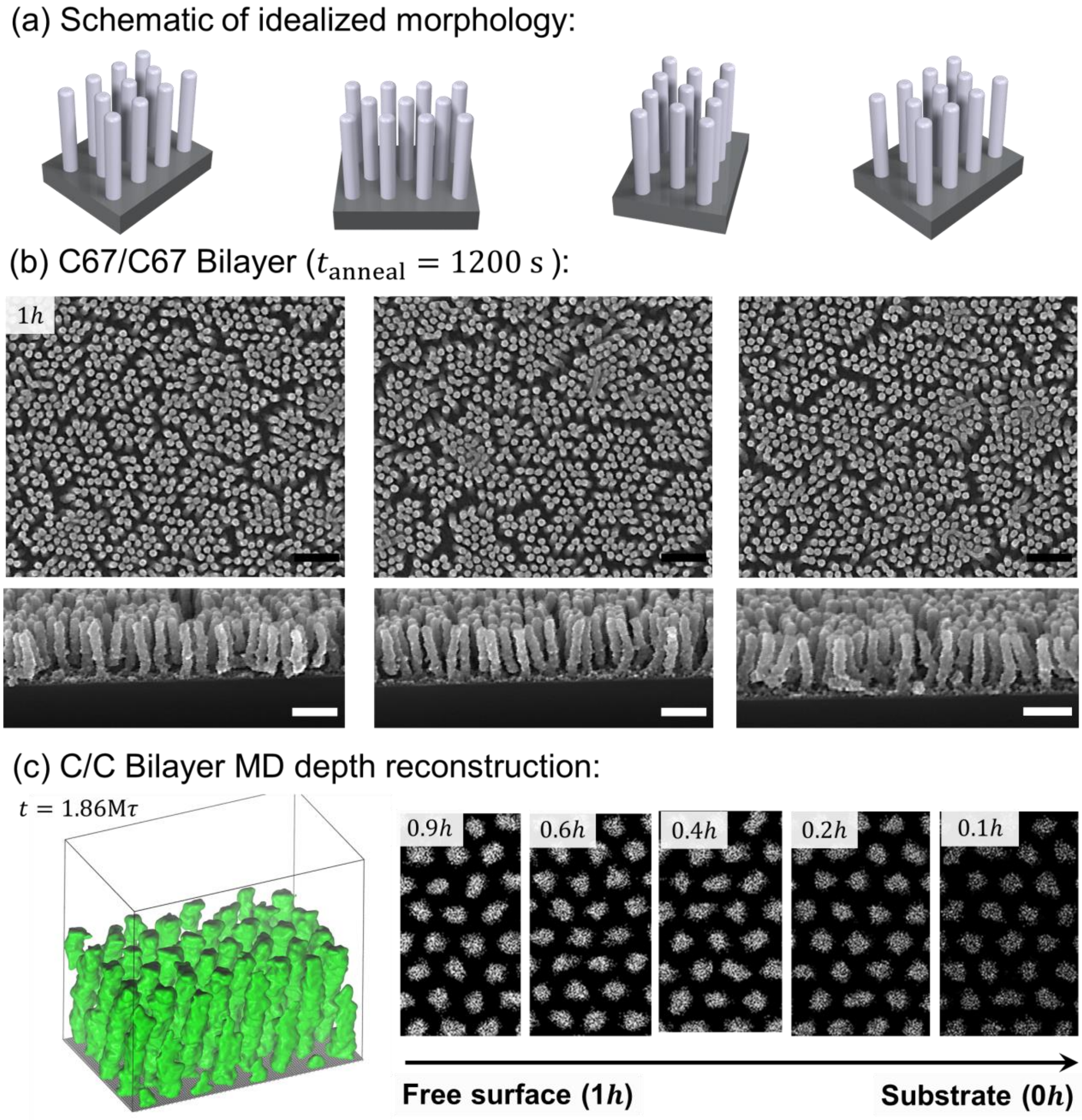

Figure 7| (a) Schematic of the vertical cylinder morphology. (b) Top down and perspective SEM images at multiple locations of a bilayer sample (C67/C67 bilayer, $t_{\text{anneal}} = 1200$ s, $\text{T} = 250\,℃$) forming the v cylinder morphology. (c) MD simulation of C/C bilayer at simulation time 1.86 Mτ; (left) perspective view and (right) planar images taken at different film depths (0*h* corresponds to substrate interface while 1*h* corresponds to film surface). All scale bars are 100 nm.

Structure e: v + h lamellae

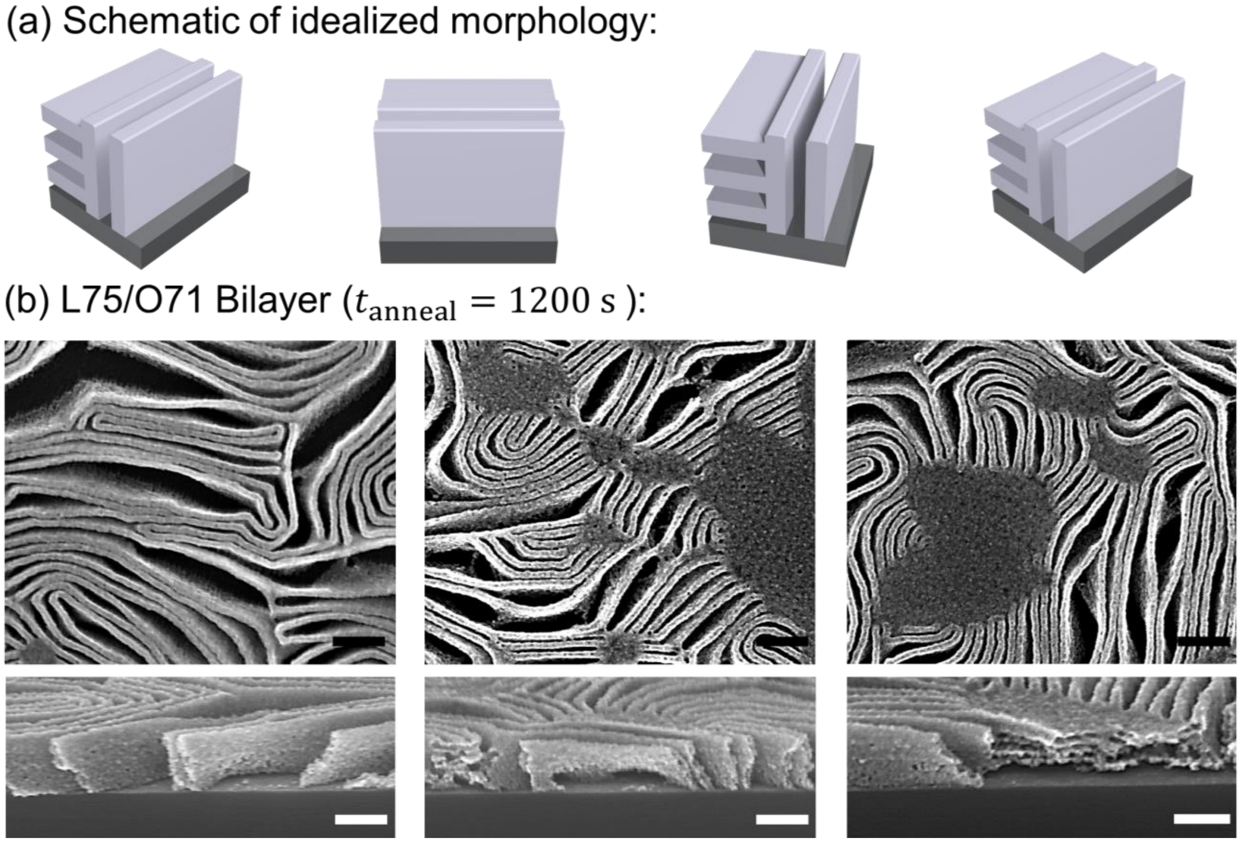

Figure 8| (a) Schematic of mixed vertical and horizontal lamellae. (b) Top down and perspective SEM images at multiple locations of a bilayer sample (L75/O71 bilayer, $t_{\mathrm{anneal}} = 1200$ s, $\mathrm{T} = 250$ ℃) forming the v+ h lamellae state. All scale bars are 100 nm.

Structure f: v lamellae

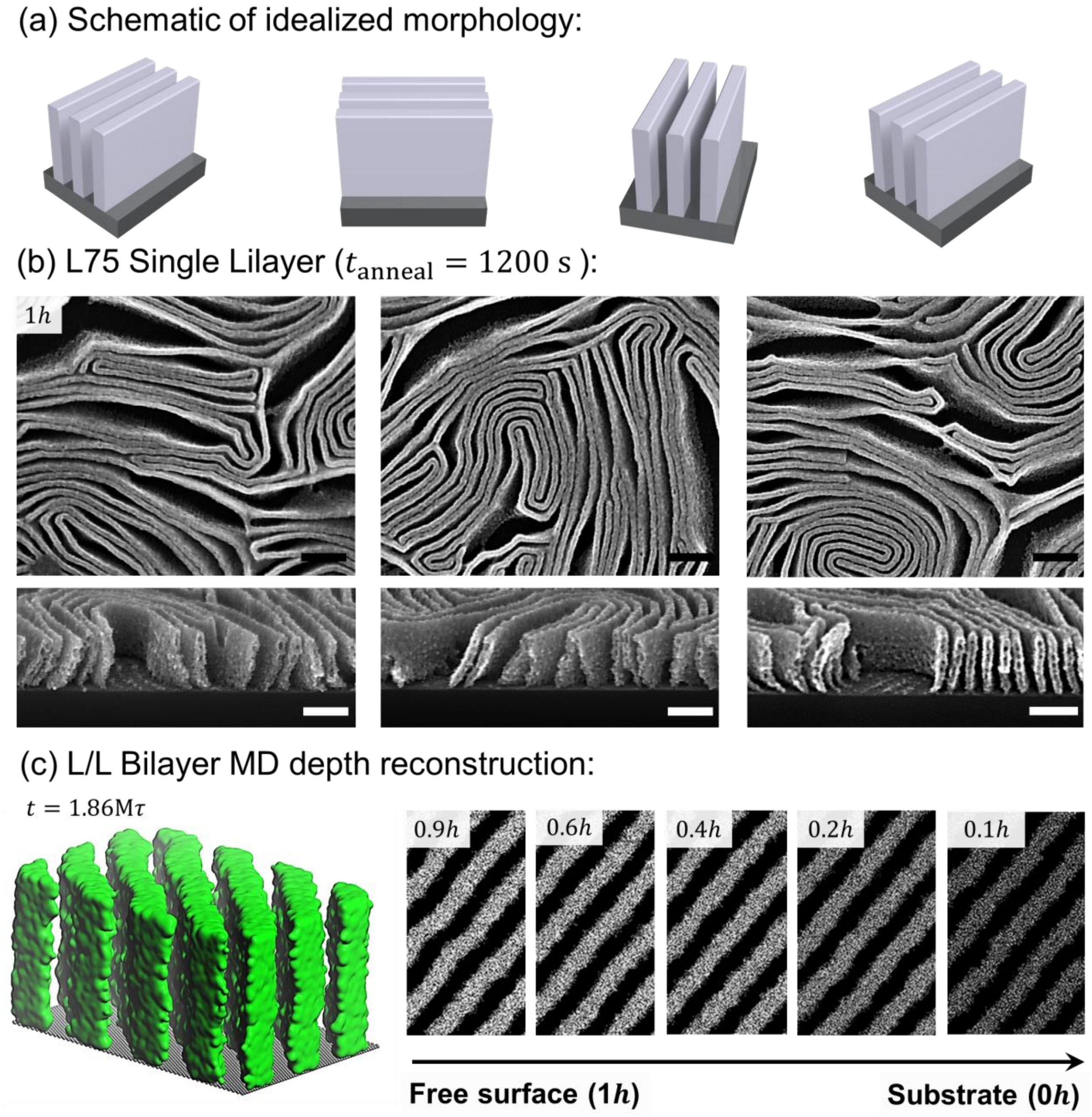

Figure 9| (a) Schematic of the vertical lamellae morphology. (b) Top down and perspective SEM images at multiple locations of a bilayer sample (L75 Single Layer, $t_{\text{anneal}} = 1200$ s, $\text{T} = 250$ ℃) forming the v lamellae morphology. (c) MD Simulation of L/L bilayer at $1.86\ \text{M}\tau$; (left) perspective view and (right) depth reconstruction. All scale bars are 100 nm.

Structure g: h inverse cylinders

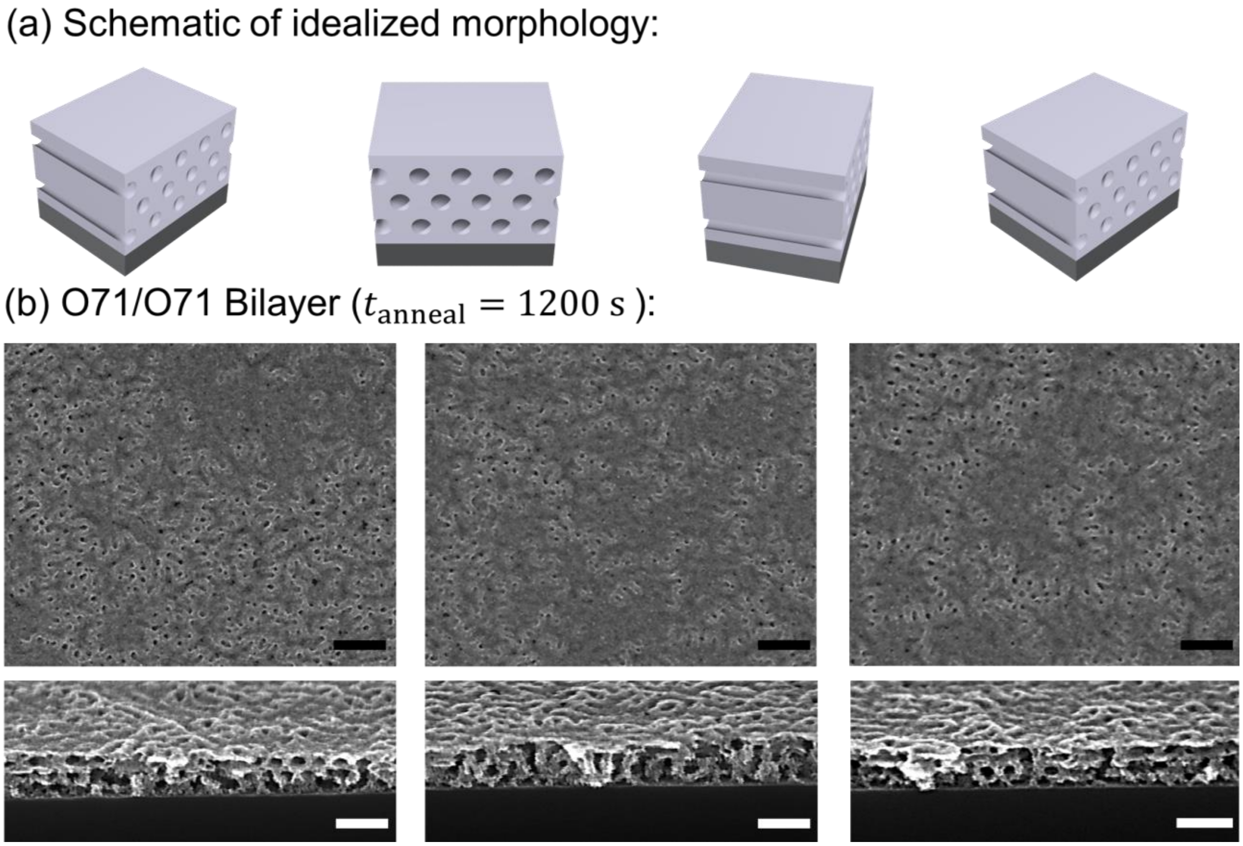

Figure 10| (a) Schematic of horizontal inverse cylinders. (b) Top down and perspective SEM images at multiple locations of a bilayer sample (O71/O71 bilayer, $t_{\mathrm{anneal}} = 1200$ s, $\mathrm{T} = 250$ ℃) forming the h inverse cylinders morphology. All scale bars are 100 nm.

Structure h: v + h inverse cylinders

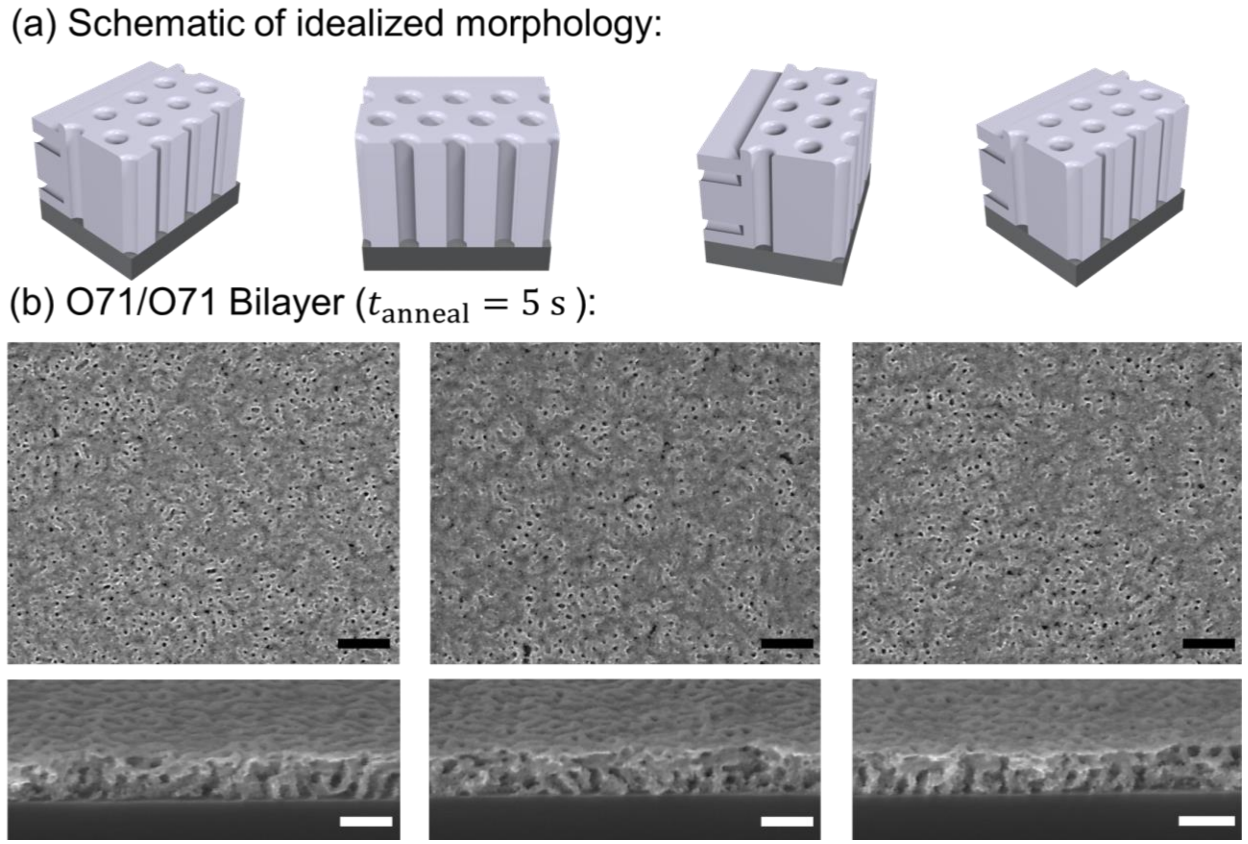

Figure 11| (a) Schematic of mixed vertical and horizontal inverse cylinders. (b) Top down and perspective SEM images at multiple locations of a bilayer sample (O71/O71 bilayer, $t_{\mathrm{anneal}} = 5$ s, $\mathrm{T} = 250$ ℃) forming the v + h inverse cylinders state. All scale bars are 100 nm.

Structure i: v inverse cylinders

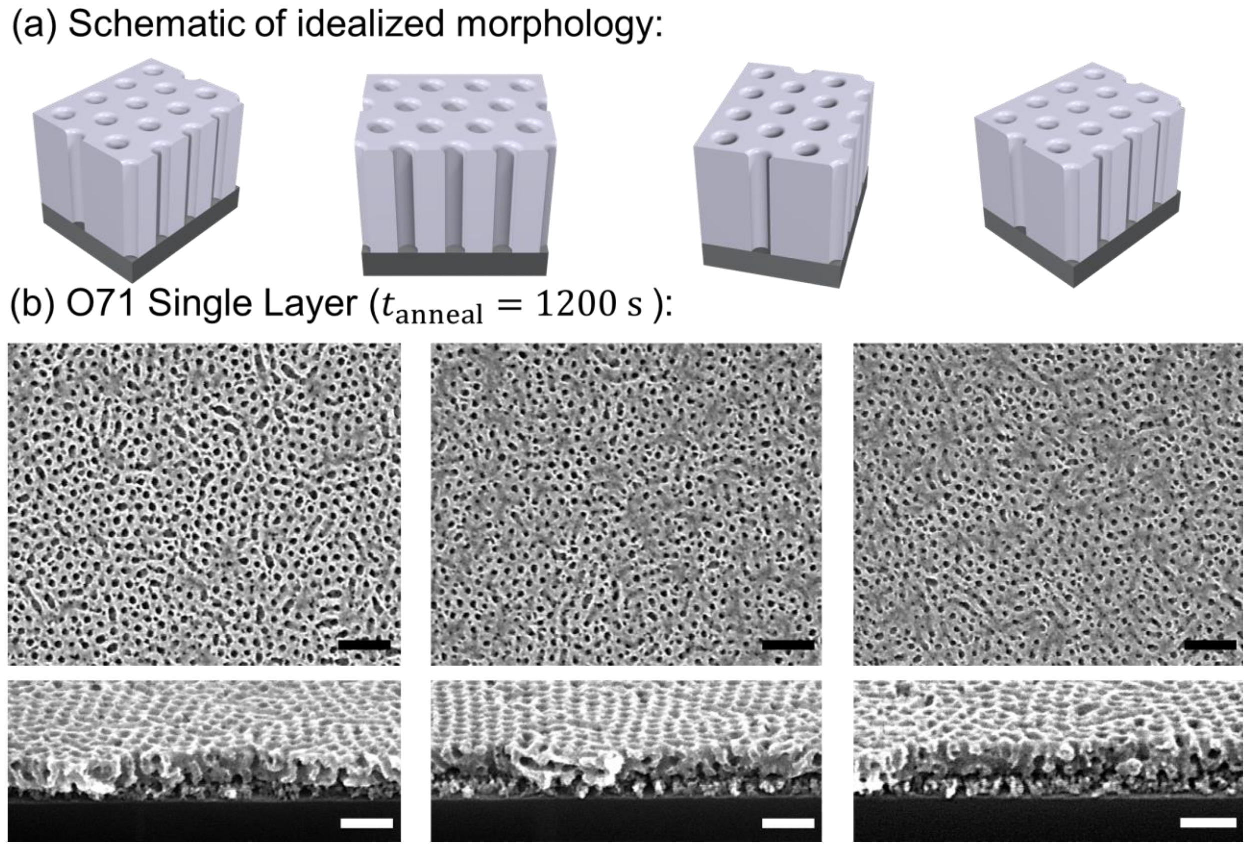

Figure 12| (a) Schematic of vertical inverse cylinders. (b) Top down and perspective SEM images at multiple locations of a bilayer sample (O71 Single Layer, $t_{\text{anneal}} = 1200$ s, $\text{T} = 250$ ℃) forming the v inverse cylinders morphology. All scale bars are 100 nm.

Structure j: h cyl-on-v lam

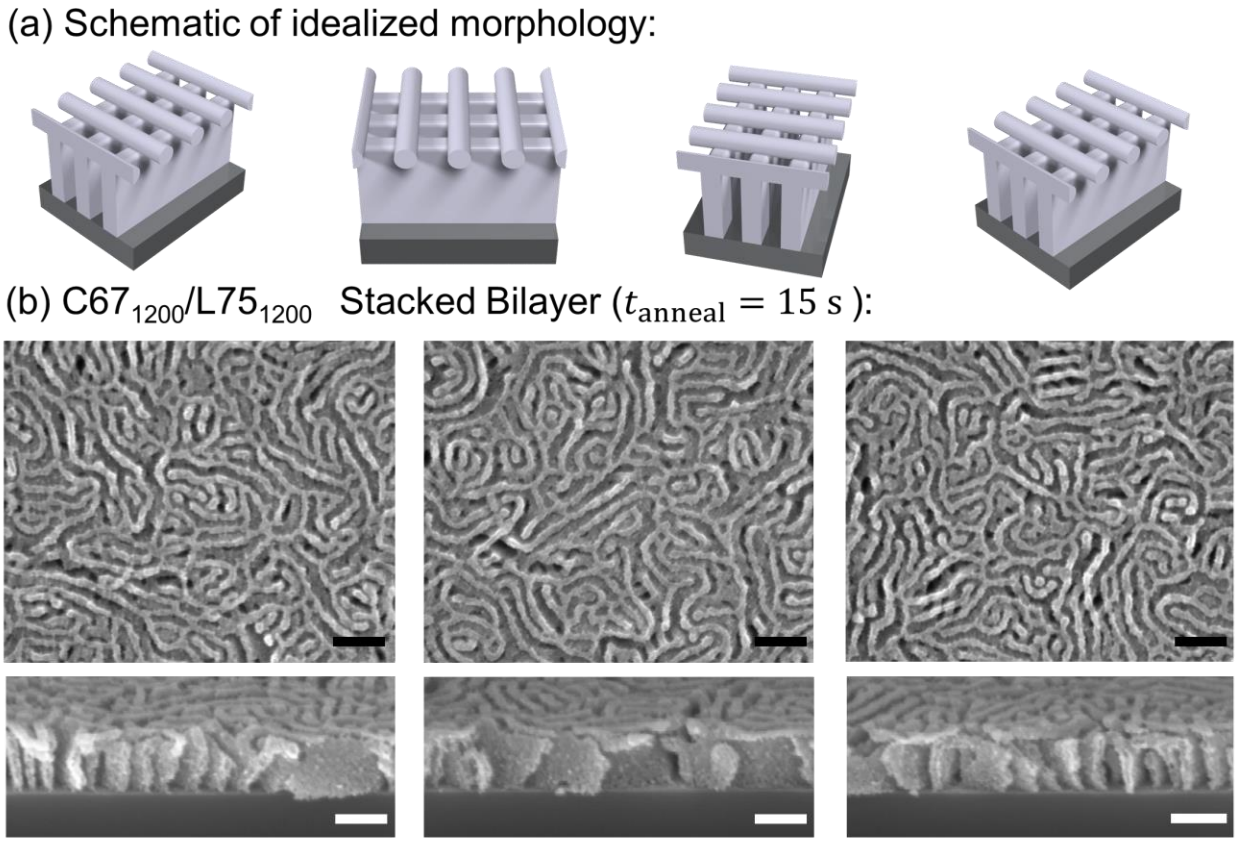

Figure 13| (a) Schematic of the identified horizontal cylinder on vertical lamellae structure. (b) Top down and perspective SEM images at multiple locations of a bilayer sample forming the h cyl-on-v lam motif. The bilayer was $C67_{1200}/L75_{1200}$, wherein each layer was independently pre-annealed (1200 s at 250 °C), and then the annealed $C67_{1200}$ layer was stacked on top of the $L75_{1200}$ layer using film transfer methods. The combined bilayer was then annealed for a further $t_{\mathrm{anneal}} = 15$ s at $\mathrm{T} = 250$ ℃. All scale bars are 100 nm.

Structure k: h HPL

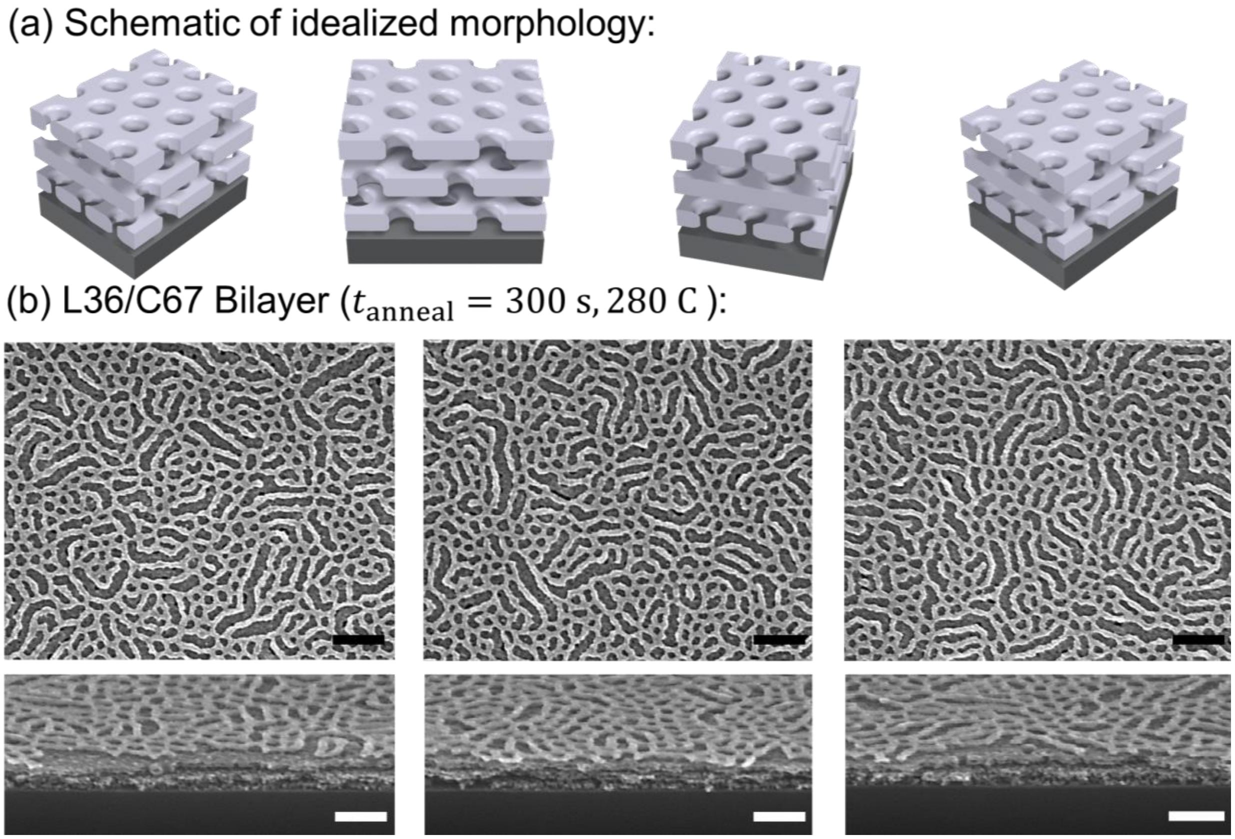

Figure 14| (a) Schematic of horizontal hexagonally perforated lamellae (HPL) morphology. (b) Top down and perspective SEM images at multiple locations of a bilayer sample (L36/C67 bilayer, $t_{\mathrm{anneal}} = 300$ s, T = 280 °C, $\phi_{\mathrm{CYL}} = 0.68$) forming a poorly-ordered h HPL. All scale bars are 100 nm.

Structure I: parapet

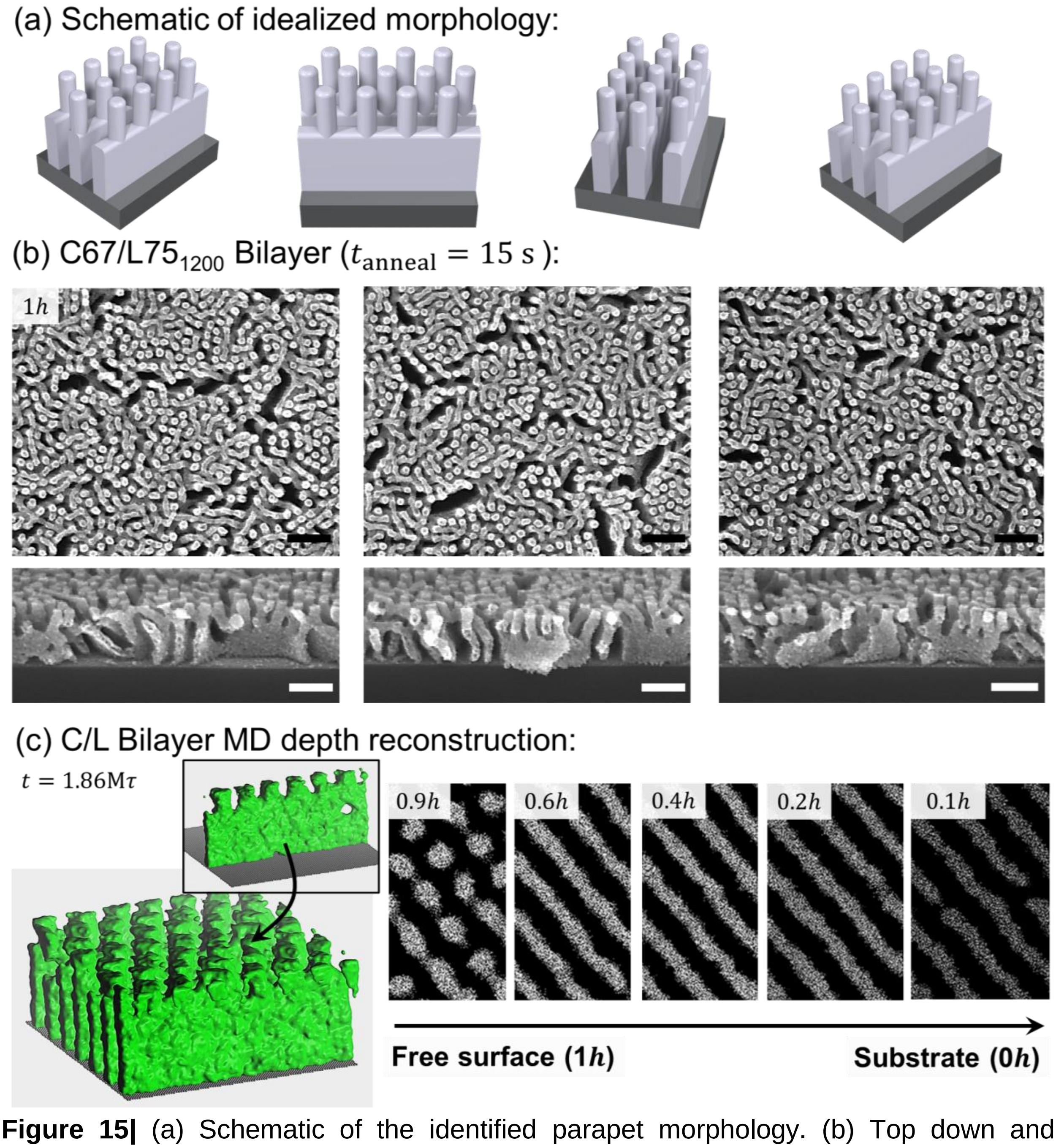

Figure 15| (a) Schematic of the identified parapet morphology. (b) Top down and perspective SEM images at multiple locations of a bilayer sample forming the parapet morphology. The bilayer was C67/L75$_{1200}$, wherein an L75 layer was pre-annealed for 1200 s at 250 °C, then a second C67 layer was cast on top using blade coating, and then combined bilayer was then further annealed for $t_{\mathrm{anneal}} = 5$ s at $\mathrm{T} = 250$ ℃. (c) MD Simulation of C/L bilayer at 0.66 Mτ; (left) perspective view and (right) depth reconstruction. All scale bars are 100 nm.

Structure m: crisscross

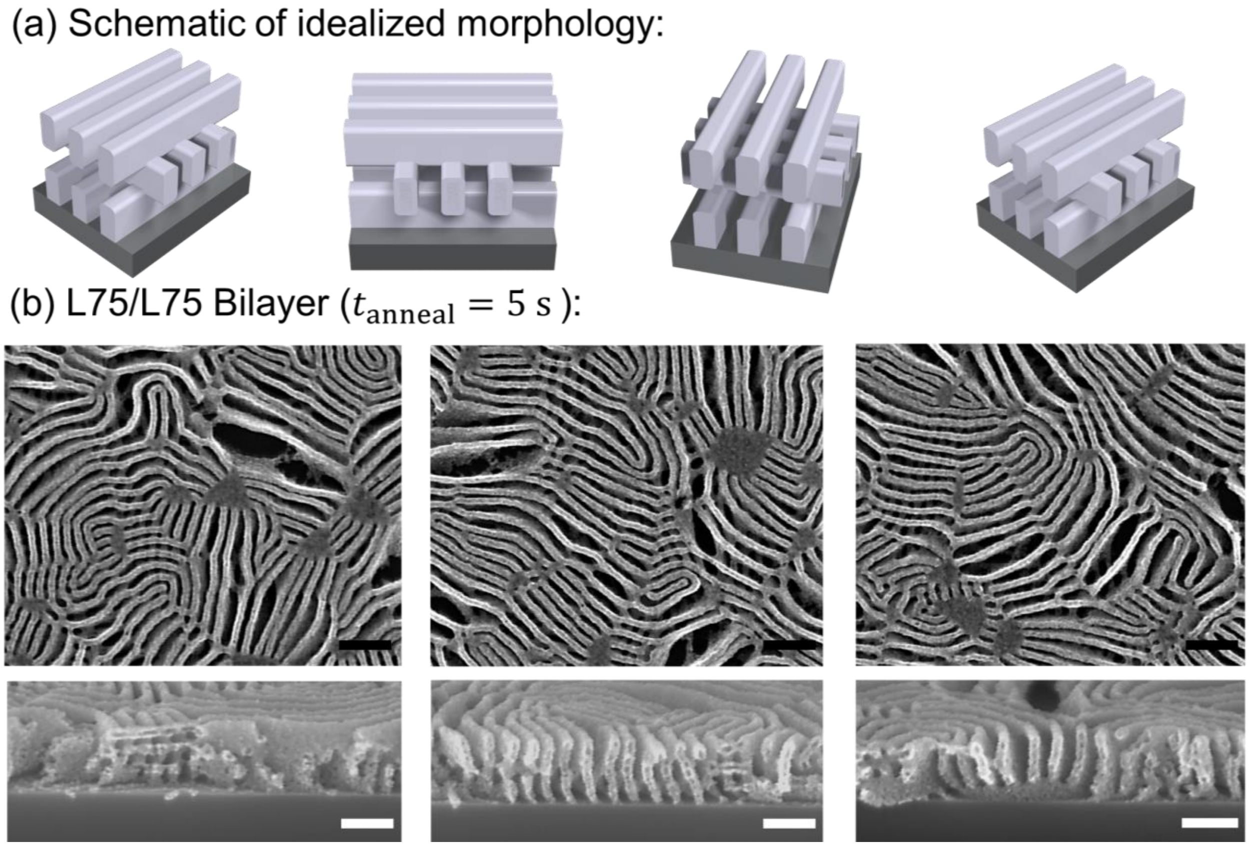

Figure 16| (a) Schematic of the identified crisscross structure. (b) Top down and perspective SEM images at multiple locations of a bilayer sample (L75/L75 bilayer, $t_{\text{anneal}} = 5$ s, $\text{T} = 250$ ℃) forming the crisscross motif. All scale bars are 100 nm.

Structure n: patchwork

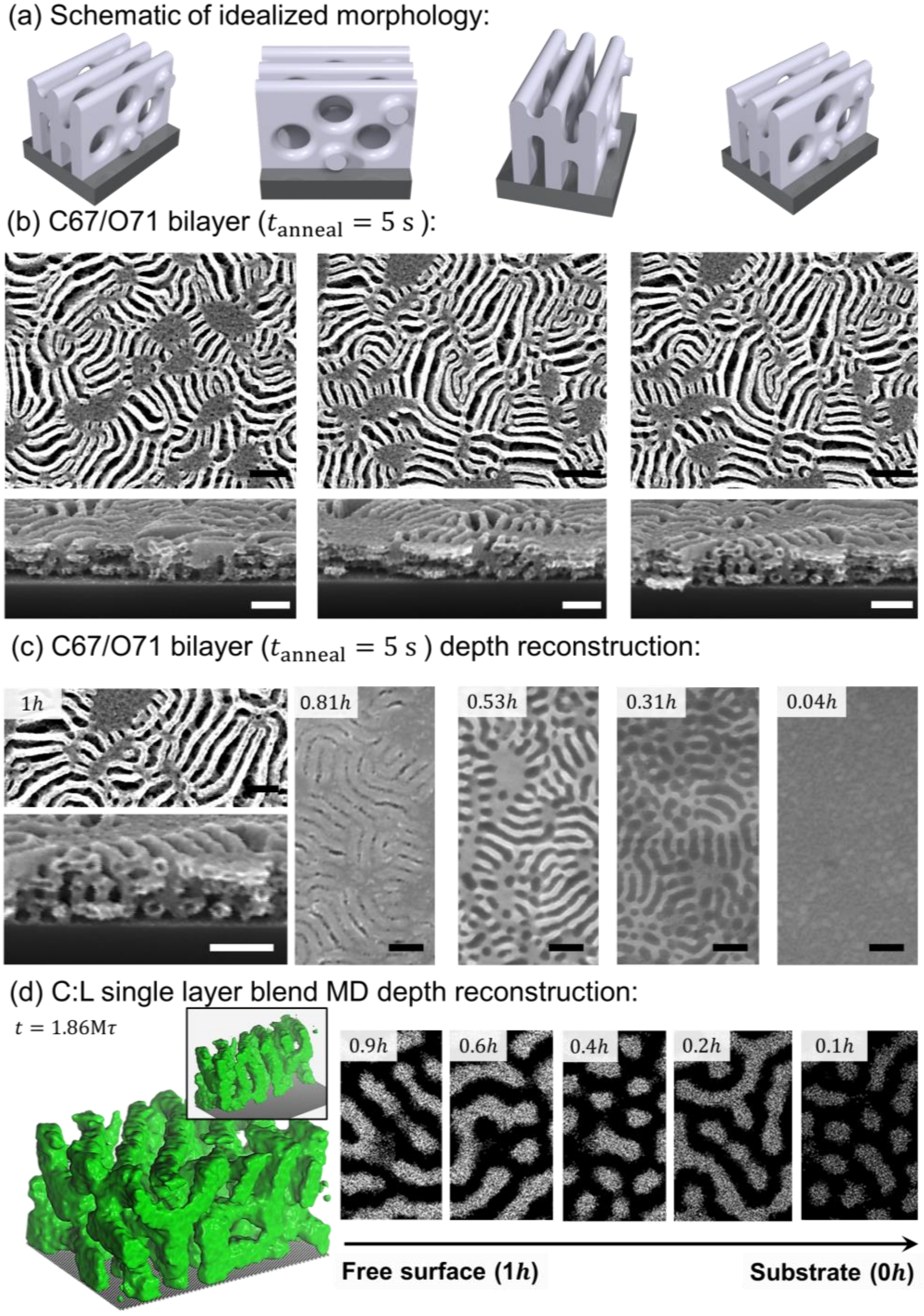

Figure 17| (a) Schematic of the patchwork motif. (b) Top down and perspective SEM images at multiple locations of a bilayer sample (C67/O71 bilayer, $t_{\text{anneal}} = 5$ s, $\text{T} = 250\ ℃$) forming the patchwork motif. (c) Top-down SEM images at various film depths ($h = 1$ and $h = 0$ are the locations of the air and substrate interfaces respectively). (c) MD simulation of C:L Blend at $0.06\ \text{M}\tau$; (left) perspective view and (right) depth reconstruction. All scale bars are 100 nm.

Structure o: v HPL

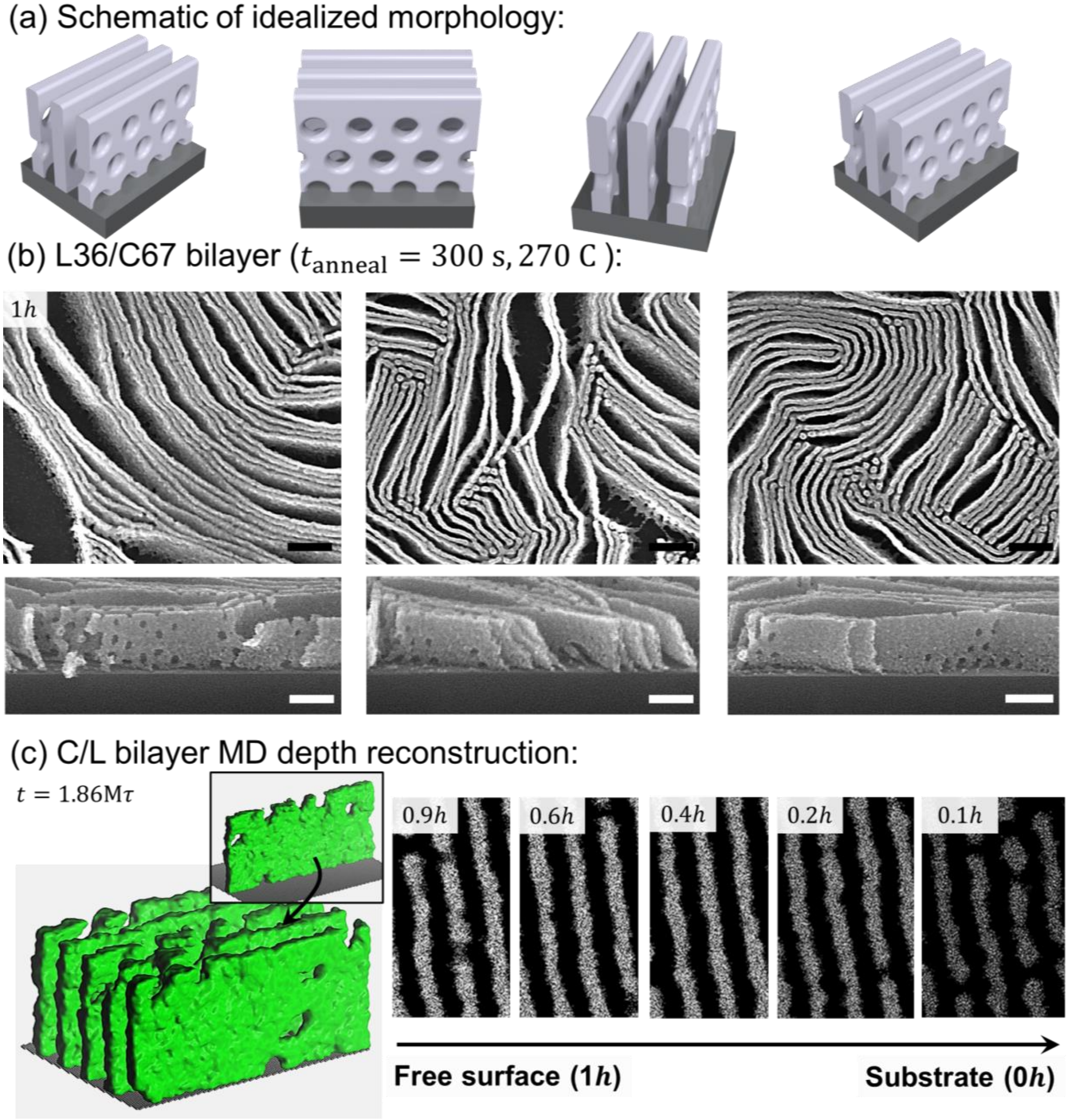

Figure 18| (a) Schematic of the vertical HPL morphology. (b) Top down and perspective SEM images at multiple locations of a bilayer sample (L36/C67 bilayer, $t_{\mathrm{anneal}} = 300$ s, $\mathrm{T} = 270$ ℃, $\phi_{\mathrm{CYL}} = 0.64$) forming the v HPL morphology. (c) MD simulation of C/L bilayer at $1.86\ \mathrm{M}\tau$; (left) perspective view and (right) depth reconstruction. All scale bars are 100 nm.

Structure p: W motif

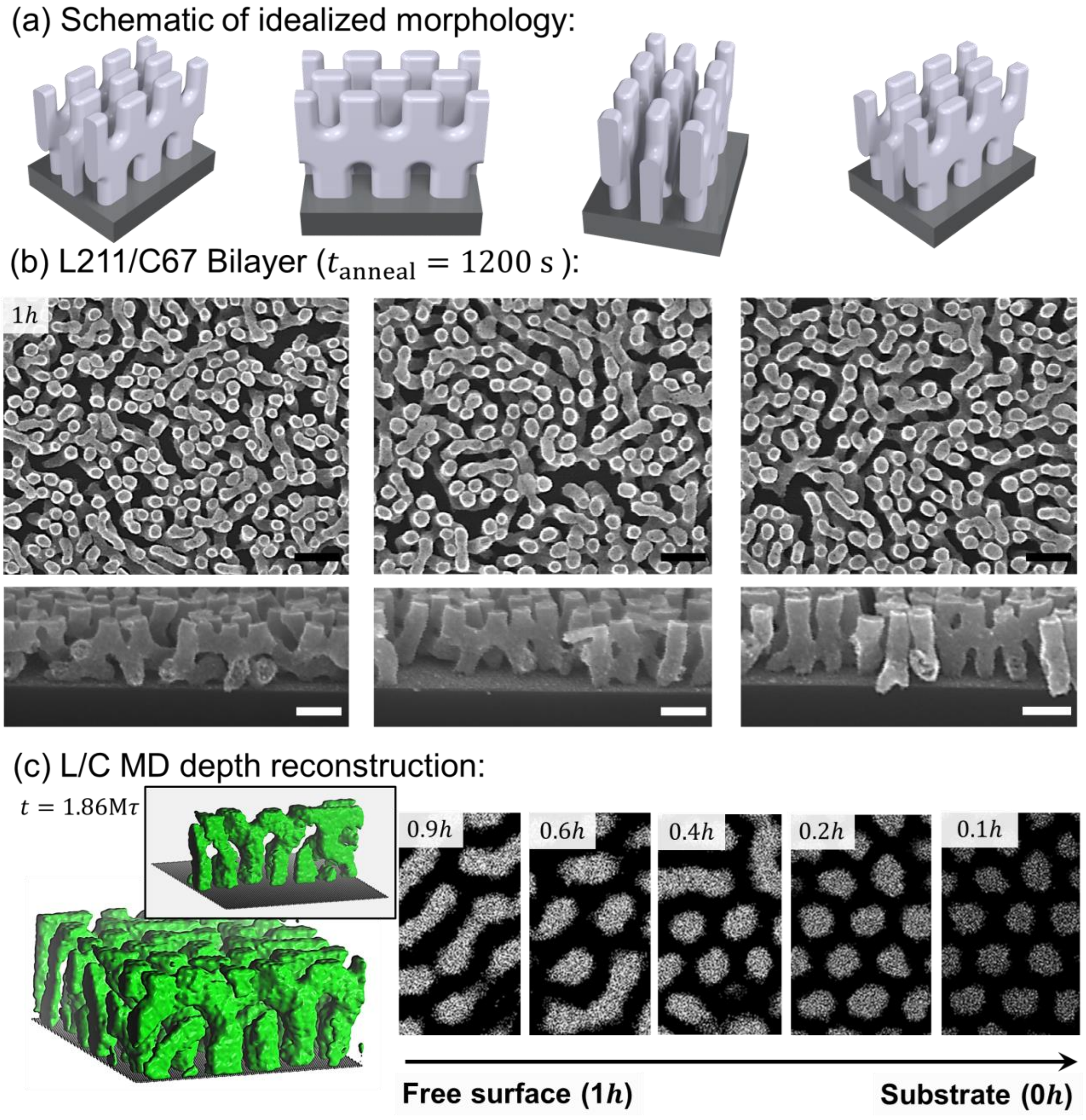

Figure 19| (a) Schematic of the identified W morphology. (b) Top down and perspective SEM images at multiple locations of a bilayer sample (L211/C67 bilayer, $t_{\text{anneal}} = 1200$ s, $\phi_{\text{CYL}} = 0.35$, $\text{T} = 250$ ℃) forming the W motif. We provide multiple locations across the sample surface to highlight that this structural motif persists across the entire samples surface. (c) MD Simulation of L/C (molecular weight of L is 50% larger than that of C) at 1.26 Mτ; (left) perspective view and (right) depth reconstruction. All scale bars are 100 nm.

Structure q: Y motif

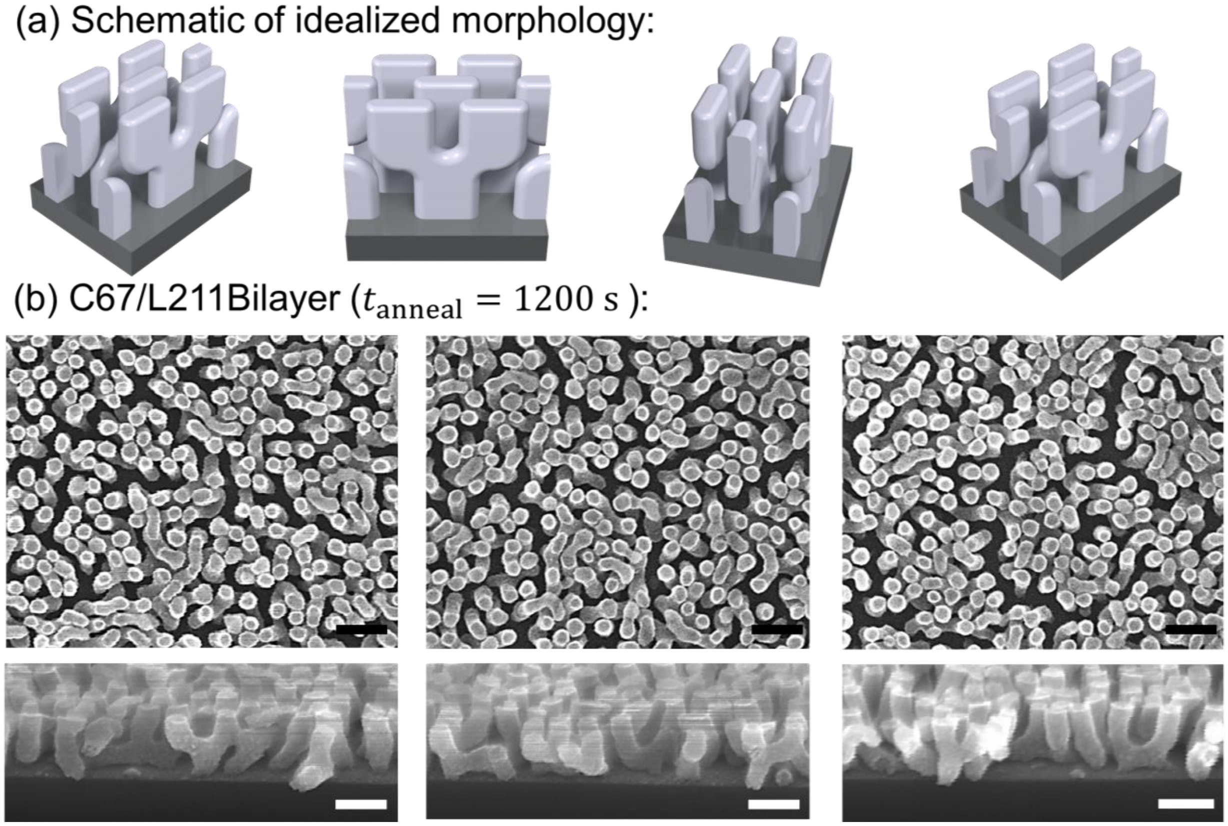

Figure 20| (a) Schematic of the identified Y morphology. (b) Top down and perspective SEM images at multiple locations of a bilayer sample (C67/L211 bilayer, $t_{\mathrm{anneal}} = 1200$ s, $\phi_{\mathrm{CYL}} = 0.46$, $\mathrm{T} = 250$ ℃) forming the Y motif. All scale bars are 100 nm.

Structure r: holes-on-lines

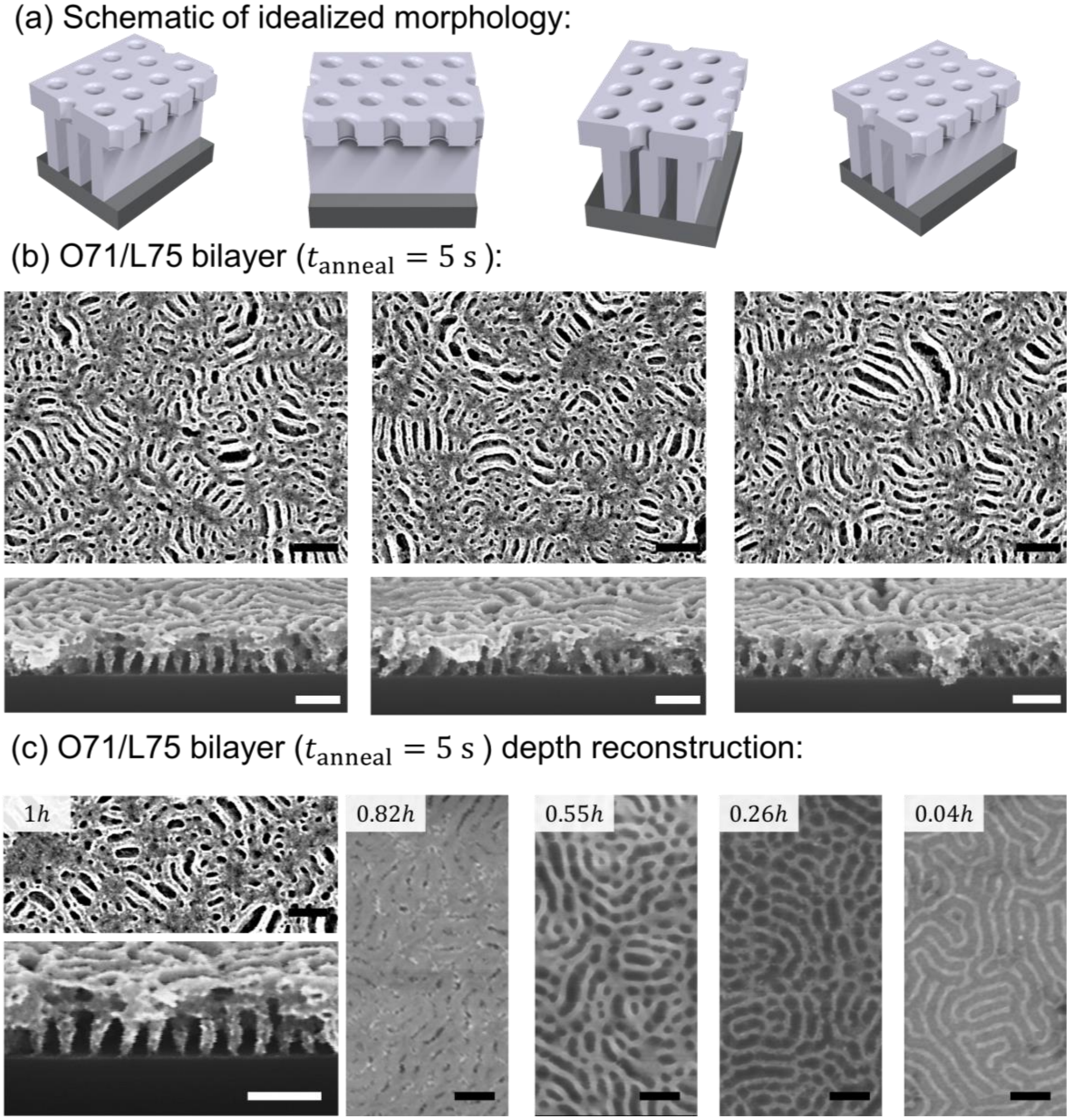

Figure 21| (a) Schematic of the holes-on-lines structure. (b) Top down and perspective SEM images at multiple locations of a bilayer sample (O71/L75 bilayer, $t_{\text{anneal}} = 5$ s, $\mathrm{T} = 250$ ℃) forming the holes-on-lines motif. (c) SEM images as a function of film depth ($h = 1$ and $h = 0$ are the locations of the air and substrate interfaces respectively). All scale bars are 100 nm.

Structure s: lines-on-holes

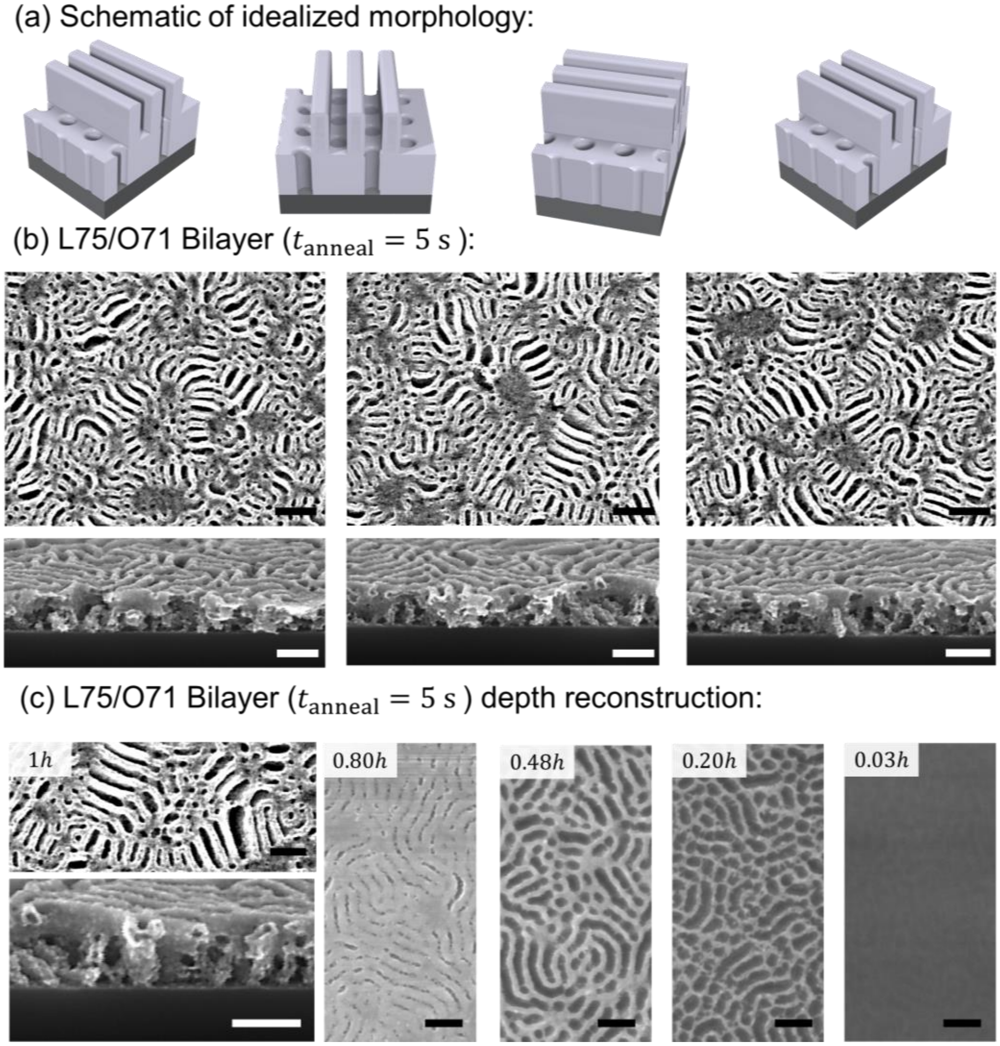

Figure 22| (a) Schematic of the lines-on-holes structure. (b) Top down and perspective SEM images at multiple locations of a bilayer sample (L75/O71 bilayer, $t_{\text{anneal}} = 5$ s, $\mathrm{T} = 250$ ℃) forming the lines-on-holes motif. (c) SEM images taken at various sample depths ($h = 1$ and $h = 0$ are the locations of the air and substrate interfaces respectively). All scale bars are 100 nm.

Structure t: footed lamellae

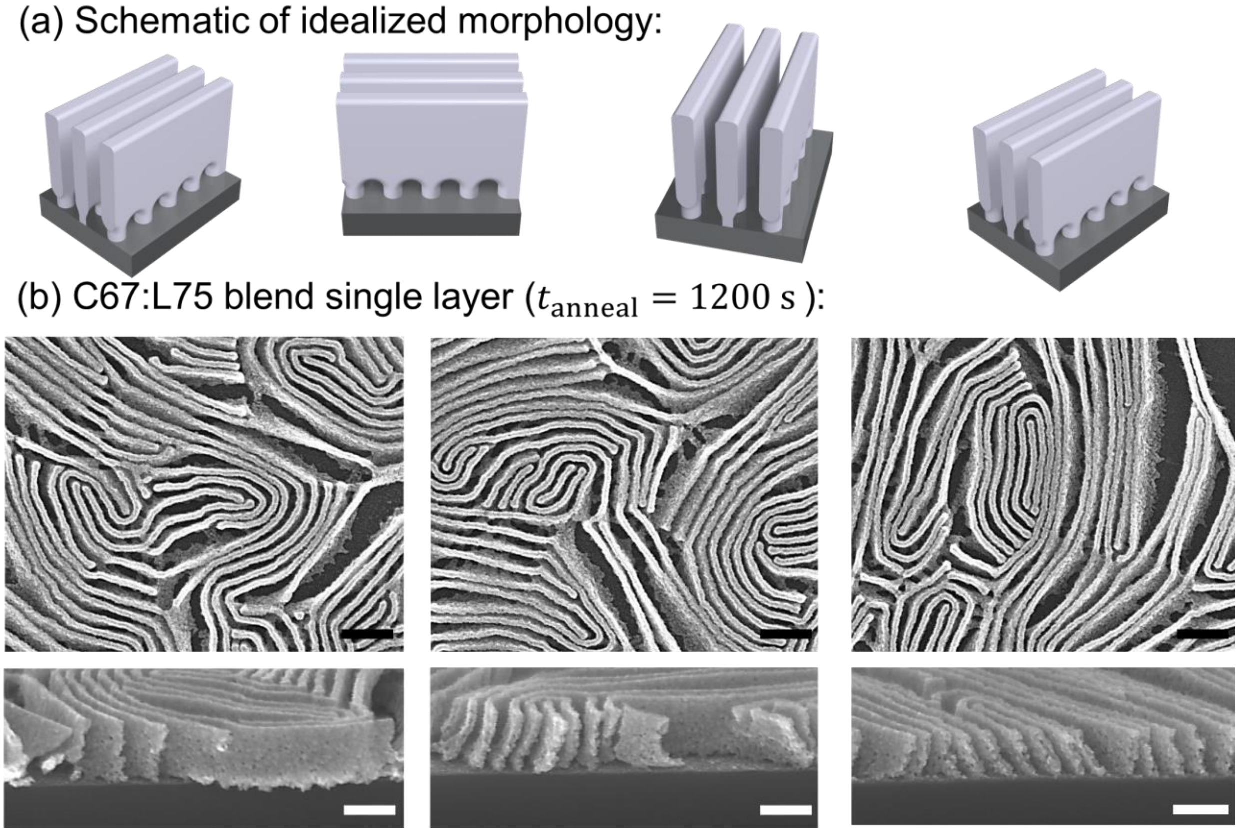

Figure 23| (a) Schematic of the identified footed lamellae morphology. (b) Top down and perspective SEM images at multiple locations of a bilayer sample (L75/C67 bilayer, $t_{\text{anneal}} = 1200$ s, $\phi_{\text{CYL}} = 0.33, \text{T} = 250\ ℃$) forming the footed lamellae morphology. All scale bars are 100 nm.

Structure u: aqueduct

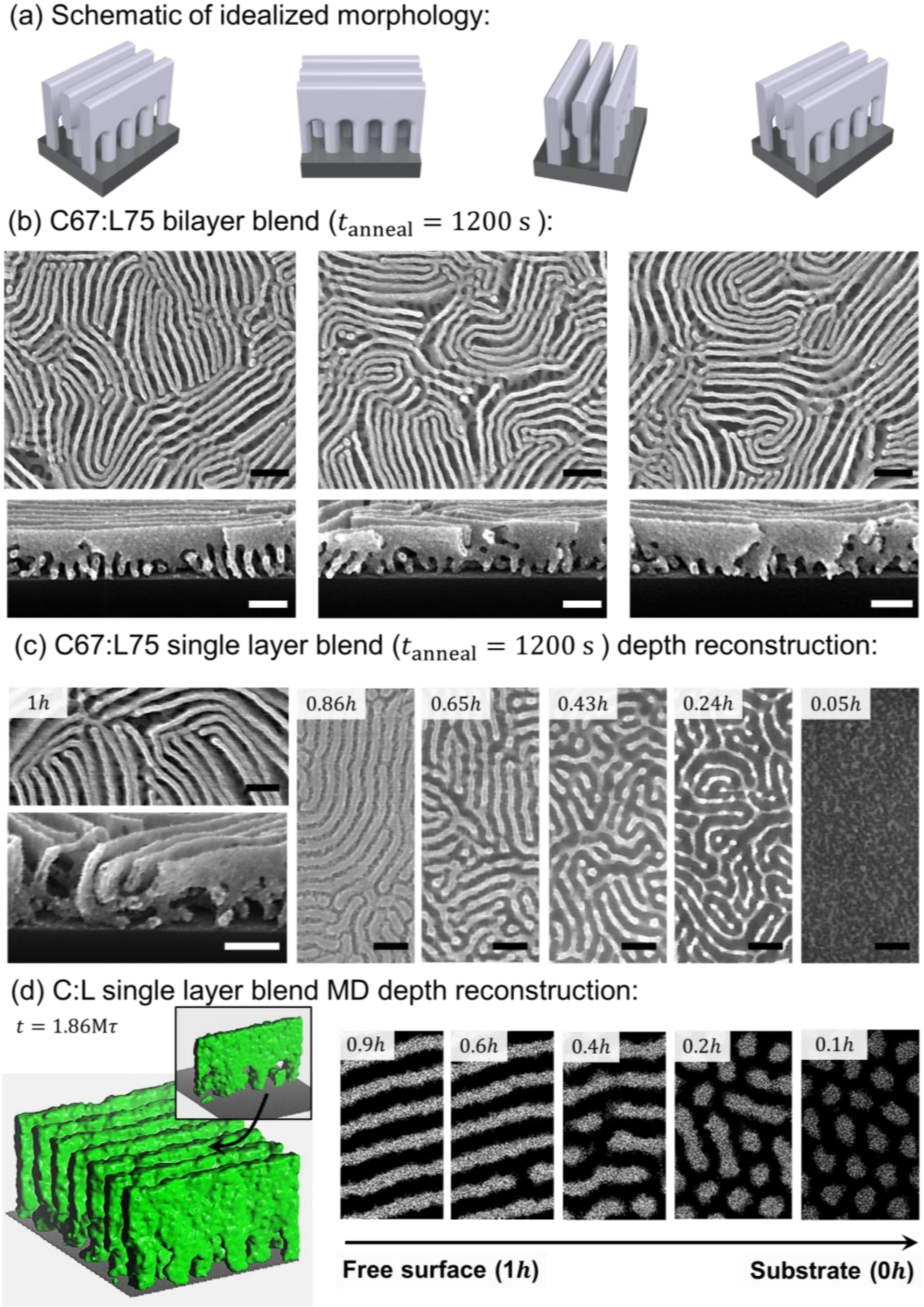

Figure 24| (a) Schematic of the identified aqueduct morphology. (b) Top down and perspective SEM images at multiple locations of a bilayer sample (C67:L75/C67:L75 bilayer blend, $t_{\text{anneal}} = 1200$ s, $\text{T} = 250$ ℃) forming the aqueduct morphology. (c) SEM images taken at various sample depths ($h = 1$ and $h =\ 0$ are the locations of the air and substrate interfaces respectively). (d) MD Simulation of L:C single layer blend at $1.86\ \text{M}\tau$; (left) perspective view and (right) depth reconstruction. All scale bars are 100 nm.

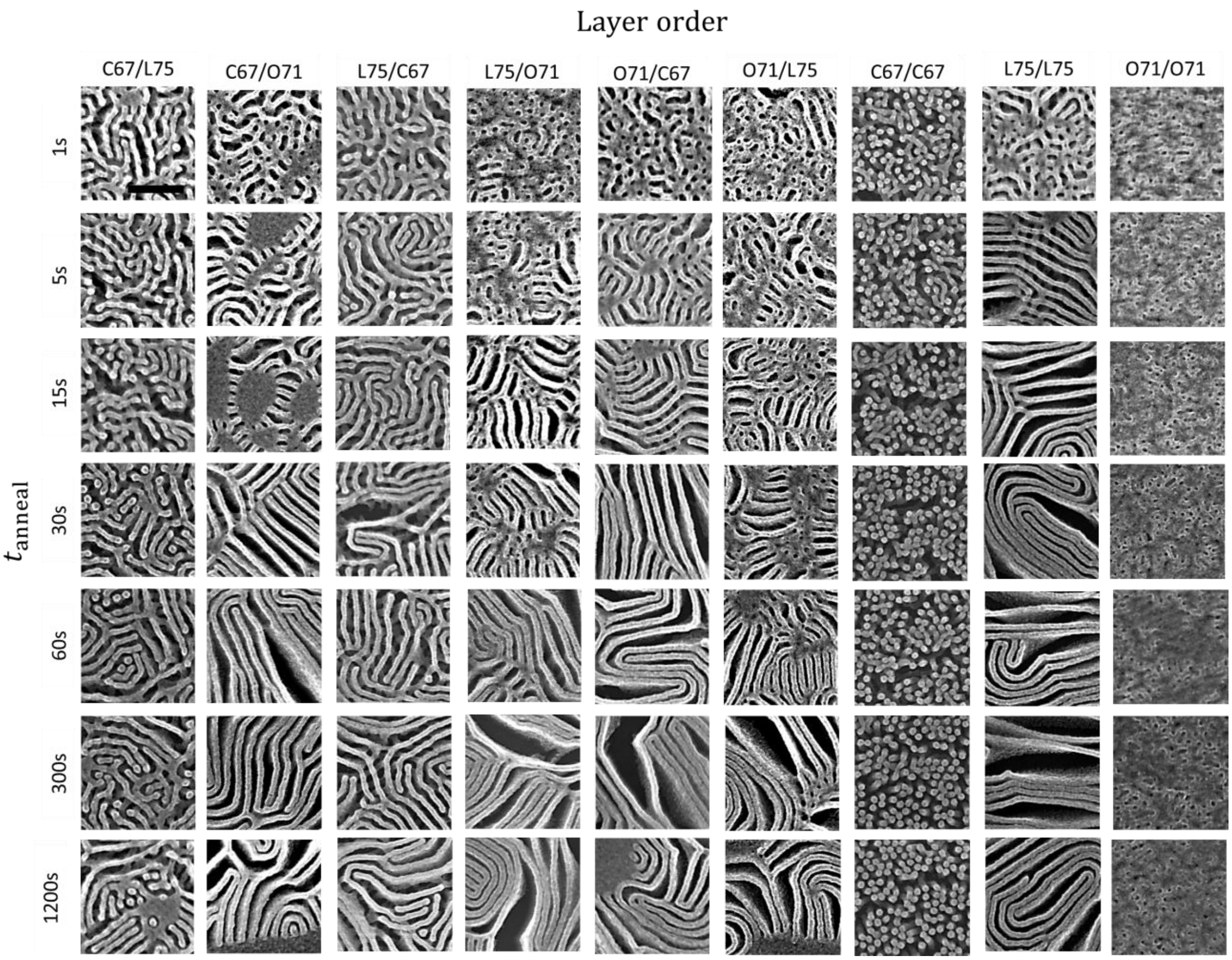

Figure 25| Top-down SEM image summary of bilayer self-assembly pathways accessible using O71, L75, and C67 and varying layer order. Each bilayer contains a 1:1 layer fraction (*e.g.* $\phi_{C67} = 0.5$ for an L75/C67 bilayer). To examine the self-assembly pathway for each bilayer many were prepared and annealed for various amounts of time (t_{anneal}, $\mathrm{T} = 250\ ℃$). Each column represents a different self-assembly pathway (changing layer order or BCP combination) and each row show all self-assembly pathways at a particular annealing time. The scale bar is 400nm and applies to all images.

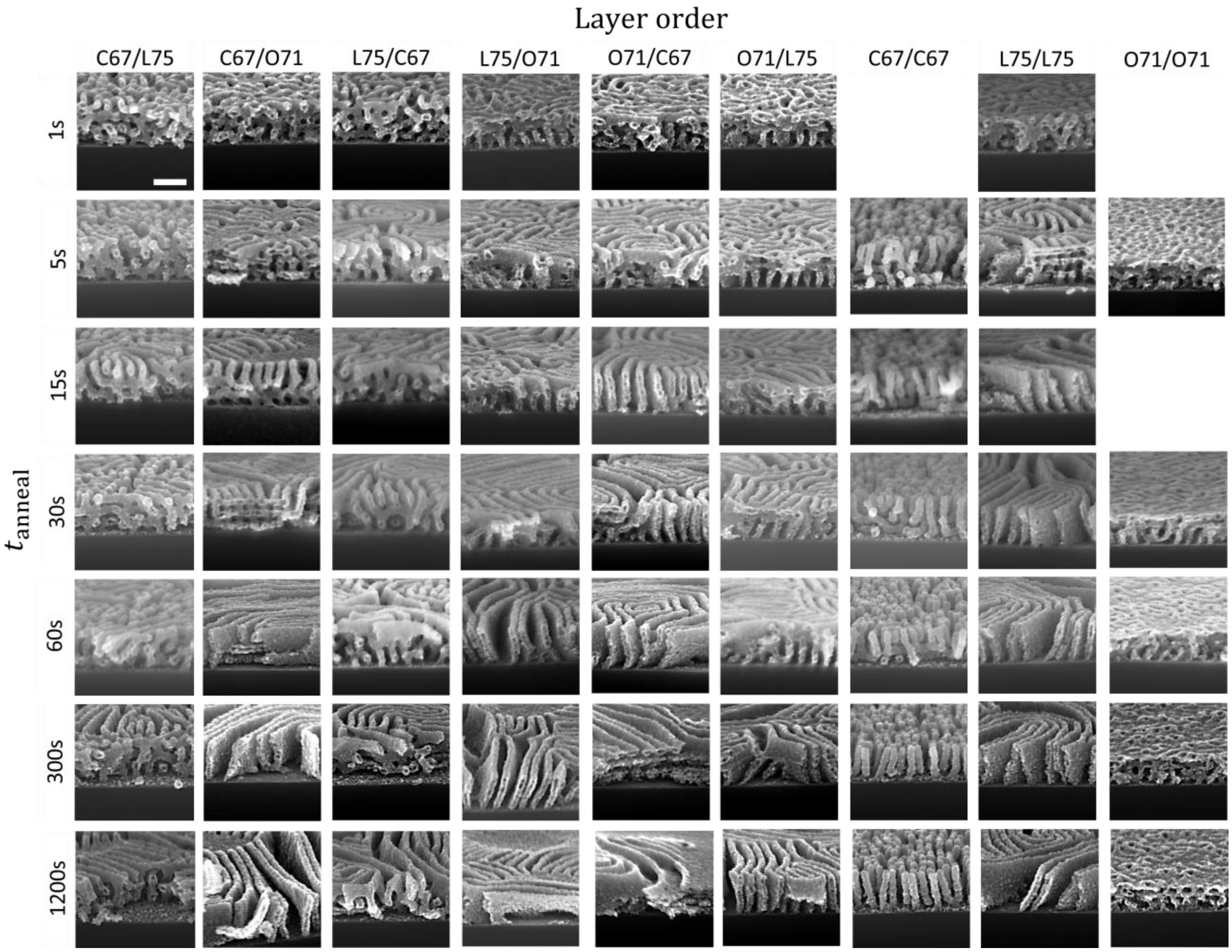

Figure 26| Cross-section SEM image summary of bilayer self-assembly pathways accessible using O71, L75, and C67 and varying layer order. Each bilayer contains a 1:1 layer fraction (*e.g.* $\phi_{C67} = 0.5$ for an L75/C67 bilayer), and was annealed at 250 °C. Each column represents a different self-assembly pathway (changing layer order or BCP combination) and each row a particular annealing time. Note the appearance of transient structures, such as crisscross (L75/L75, $t_{anneal} = 5$ s , $\mathrm{T} = 250$ ℃) and holes-on-lines (O71/L75, $t_{anneal} = 5$ s, $\mathrm{T} = 250$ ℃); stable non-native morphologies at long anneal times (aqueduct, C67/L75 & L75/ C67, $t_{anneal} = 1200$ s, $\mathrm{T} = 250$ ℃); and kinetically trapped states at long anneal times (*e.g.* O71/C67, $t_{anneal} = 1200$ s, $\mathrm{T} = 250$ ℃ – vertical + horizontal lamellae).The scale bar is 400nm.

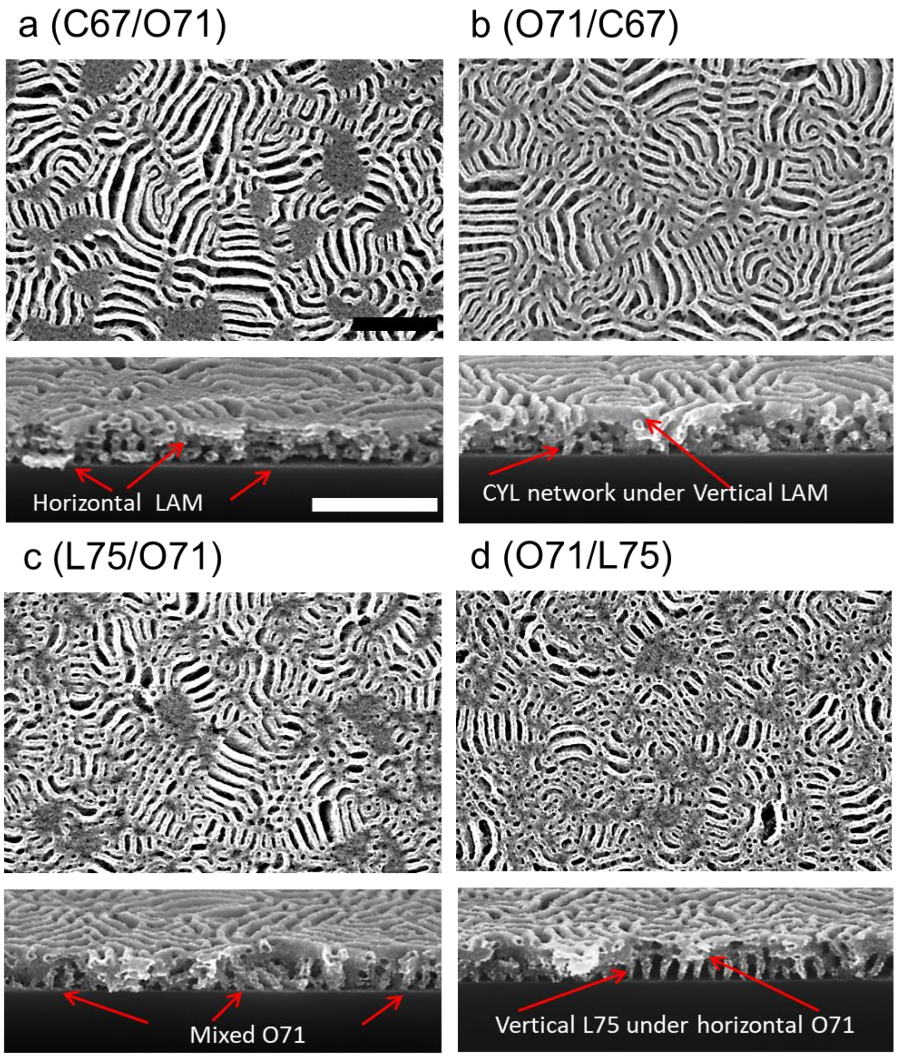

Figure 27| Impact of layering order on a variety of possible bilayer combinations. Each sample was annealed for 5 s at $\mathrm{T} = 250\ ^{\circ}\mathrm{C}$ to observe the transient states that occur during the early stages of self-assembly, where the layering order is expected to have an influence on the BCP morphology. (a) Top down and cross-sectional SEM of C67 layered on O71 display a patchwork of mixed lamellae orientations located at different parts of the film (top and bottom as noted on the cross-section). (b – d) SEM images of O71/C67, L75/O71, and O71/L75 bilayers respectively. The top-down SEM shows a mixture of various motifs (lines, inverse cylinders – where PMMA is the matrix, and horizontal lamellae) on top of a network of (b) cylindrical structures, (c) inverse cylinders, and (d) vertical lamellae. The scale bars are 300 nm and apply to all images.

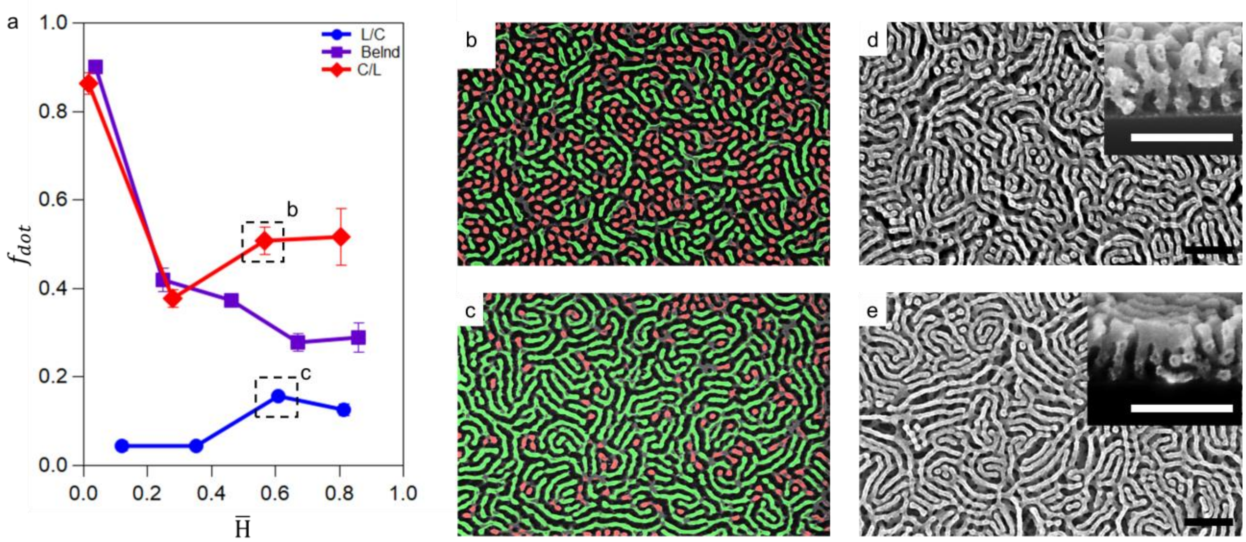

Figure 28| From strictly examining the top-down and cross-section SEM images it is not immediately obvious what structural features differentiate C/L (C67/L75) and L/C (L75/C67) bilayers ($\phi_{C67} = 0.5$) films from each other and the blend (1:1 C67:L75 blend) after 5s of thermal annealing. To elucidate their differences, we used O_2 plasma etching in combination with image analysis to extract structural metrics (domain spacing, structural correlation lengths, defect density, and dot fraction) as a function of relative film thickness ($\overline{\mathrm{H}} = 1 - \mathrm{h_e}/\mathrm{h_{total}}$, where $\mathrm{h_e}$ and $\mathrm{h_{total}}$ are the etch depth distance and total film thickness respectively) of each film. Note that the substrate and air interfaces is located at $\overline{\mathrm{H}} = 0$ and $\overline{\mathrm{H}} = 1$ respectively. (a) Plot of the dot area fraction (f_{dot}) as a function of $\overline{\mathrm{H}}$. (b, c) Examples of f_{dot} determination for C67/L75, blend, and L75/C67 bilayer films annealed for 5 s respectively. The differentiation of dots (red) and lines (green) generated by the image analysis is overlaid on the actual SEM image. (d, e) Top-down SEM images with cross-section insets of C67/L75, blend, and L75/C67 bilayer films annealed for 5s at $\mathrm{T} = 250\ ℃$. All scale bars are 200nm.

Experimental data for BCP bilayers with molecular weight disparity

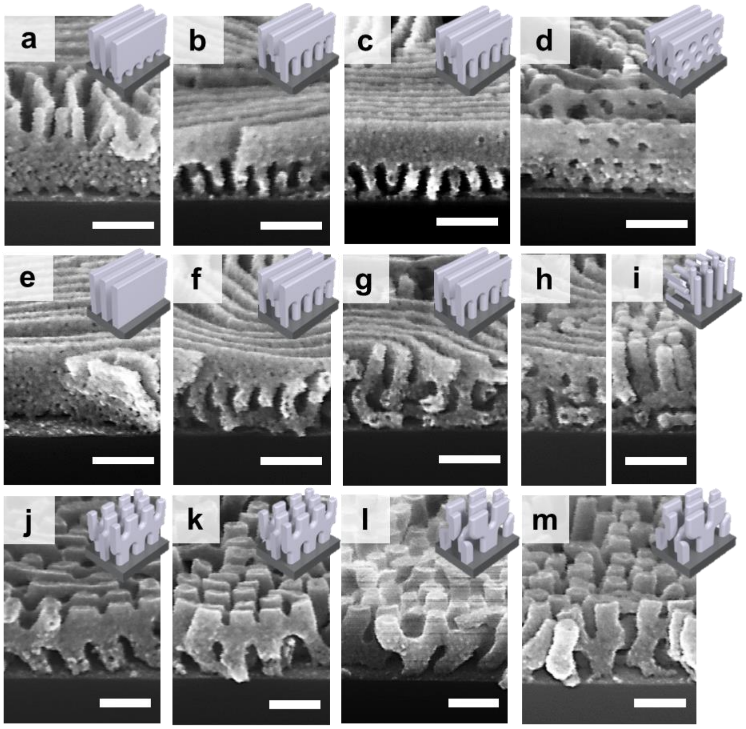

Figure 29| Non-native motifs driven by molecular weight disparity. The cross-sectional SEM images (a-m) show representative non-equilibrium structures forming in different molecular weight disparity ($N_{\mathrm{CYL}}/N_{\mathrm{LAM}}$) regimes for $t_{\mathrm{anneal}} = 300\,\mathrm{s}$. (a – d) Bilayers with $N_{\mathrm{CYL}}/N_{\mathrm{LAM}} \gg 1$ were constructed using L36 and C67 BCPs. Increasing the ϕ_{CYL} yields different non-native morphologies such as footed lamellae (a: L37/C67, $\phi_{\mathrm{CYL}} = 0.35$) and aqueduct (b: L37/C67, $\phi_{\mathrm{CYL}} = 0.46$, c: L37/C67, $\phi_{\mathrm{CYL}} = 0.54$) while changing the annealing temperature revealed the v-HPL morphology (d: L37/C67, $\phi_{\mathrm{CYL}} = 0.64, \mathrm{T} = 270℃$) suggesting another experimental axis to explore. (e – i) Bilayers with $N_{\mathrm{CYL}}/N_{\mathrm{LAM}} \sim 1$ were construct using L75 and C67 BCPs. Increasing the ϕ_{CYL} yields different non-native morphologies such as footed lamellae (e: L75/C67, $\phi_{\mathrm{CYL}} = 0.33$) and aqueduct (f: C67/L75, $\phi_{\mathrm{CYL}} = 0.38$, g: C67/L75, $\phi_{\mathrm{CYL}} = 0.45$) while changing the layer order can produce subtle morphological defects (h: L75/C67, $\phi_{\mathrm{CYL}} = 0.59$, i: C67/L75, $\phi_{\mathrm{CYL}} = 0.56$). (j – m) Bilayers with $\mathrm{N_{CYL}/N_{LAM}} \ll 1$ were constructed using L211 and C67 BCPs. Increasing the ϕ_{CYL} yields different non-native motifs such as W motif (j: L211/C67, $\phi_{\mathrm{CYL}} = 0.31,$ k: L211/C67, $\phi_{\mathrm{CYL}} = 0.35$) and the Y motif (l: C67/L211 $\phi_{\mathrm{CYL}} = 0.38$, m: L211/C67 $\phi_{\mathrm{CYL}} = 0.45$). All self-assemblies were performed on Si substrates functionalized with random copolymer brush (61 mol% PS) and annealed at $\mathrm{T} = 250\ ℃$ unless specified otherwise. All scale bars are 100 nm.

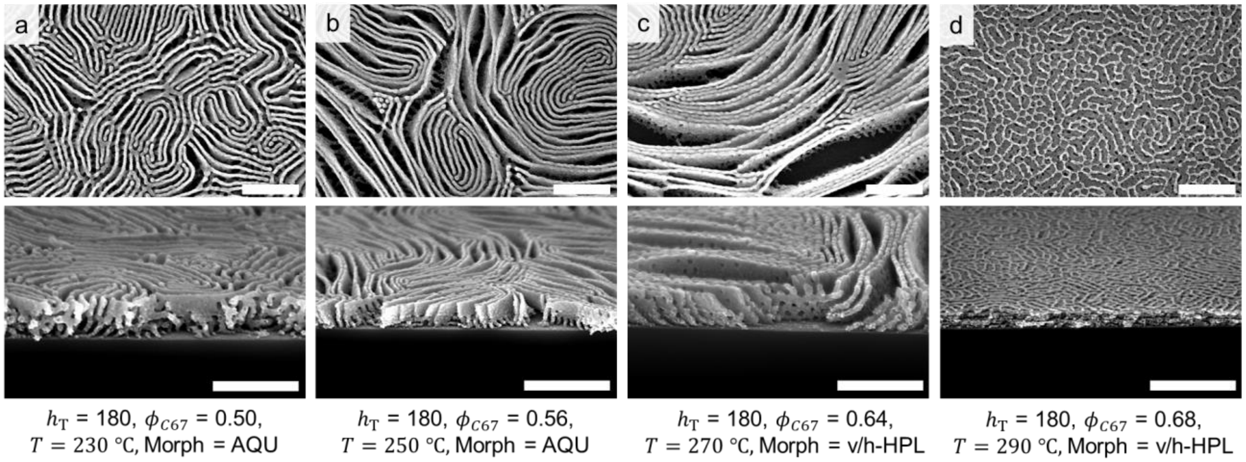

Figure 30| (a – d) Top down and cross-sectional SEM images of L36 on C67 bilayer (L36/C67) annealed at various temperatures (*T*). Using the top-down and cross-sectional SEM images each morphology was label as either AQU = aqueduct, v/h-HPL = mixture of vertically and horizontally oriented perforated lamellae, and h-HPL = horizontally oriented perforated lamellae. All scale bars are all 300nm.

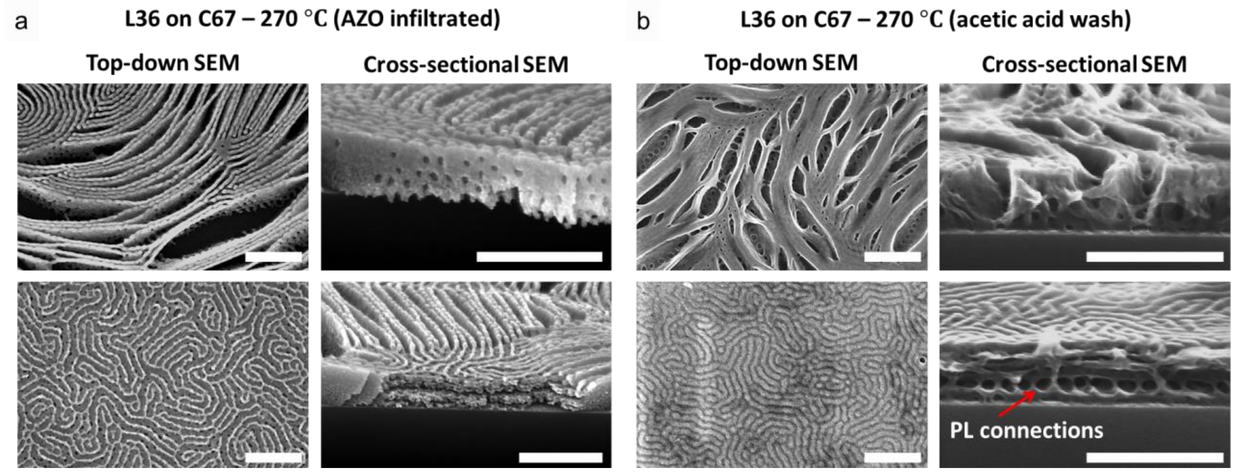

Figure 31| (a) SEM images of AZO replicas, and (b) UV/acetic acid treated L36/C67 bilayer annealed at 270 ℃.Two different protocols film treatment protocols and both show the perforated lamellae morphology, confirming the structure. All scale bars are 300nm.

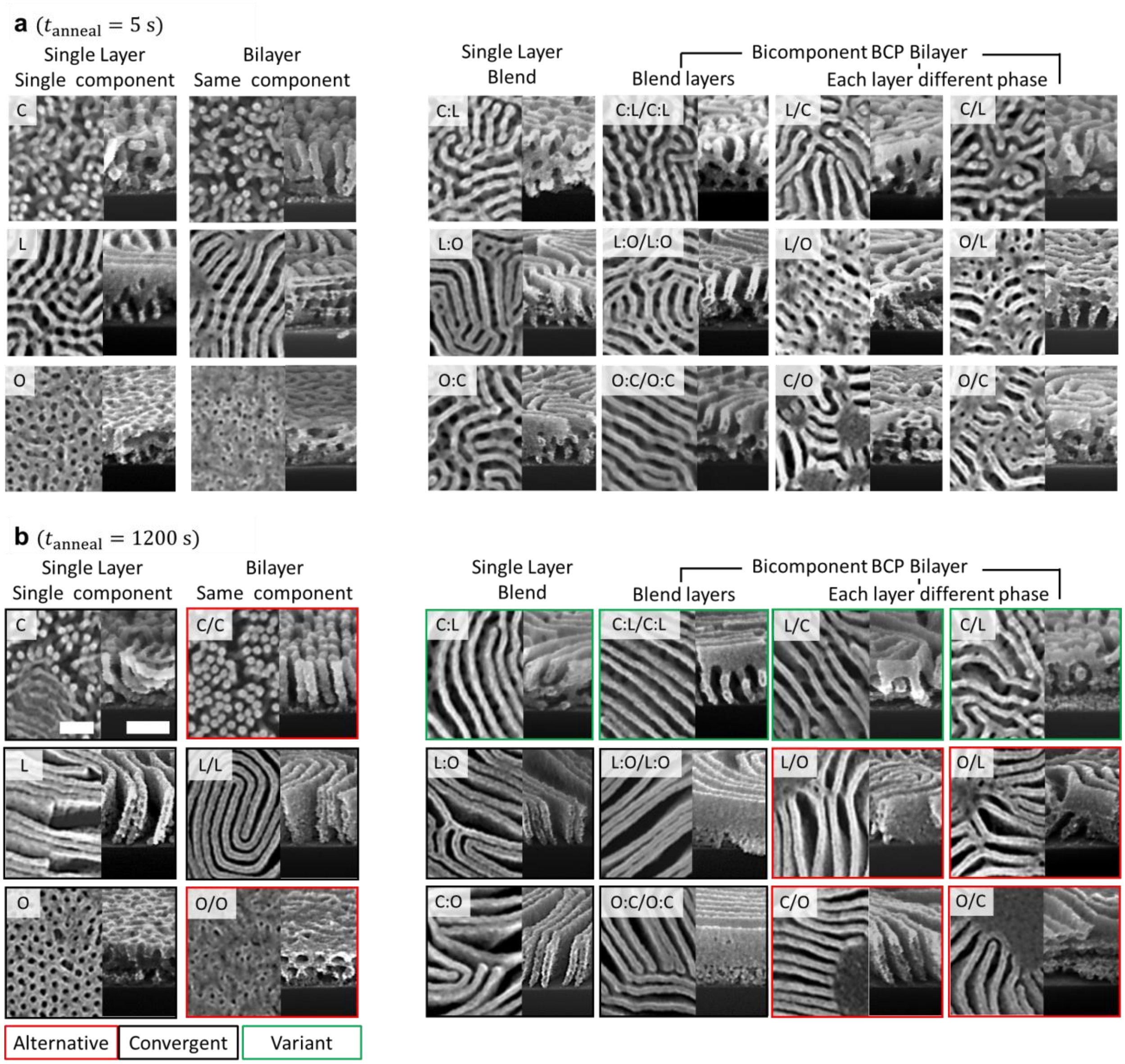

Figure 32| SEM images (top down and cross-section) of same-component and bi-component BCP bilayers compared their single layer analogs annealed at 250°C for (a) 5 s and (b) 1200 s. Different evolution behaviors can be observed (denoted by color of outline box): some pathways converge together into a common final morphology (black boxed), while other pathways lead to variants of a particular motif (green boxed). Some self-assembly pathways become kinetically trapped in metastable states (red boxed). Scale bars are 100nm.

Experimental data for electrical characterization

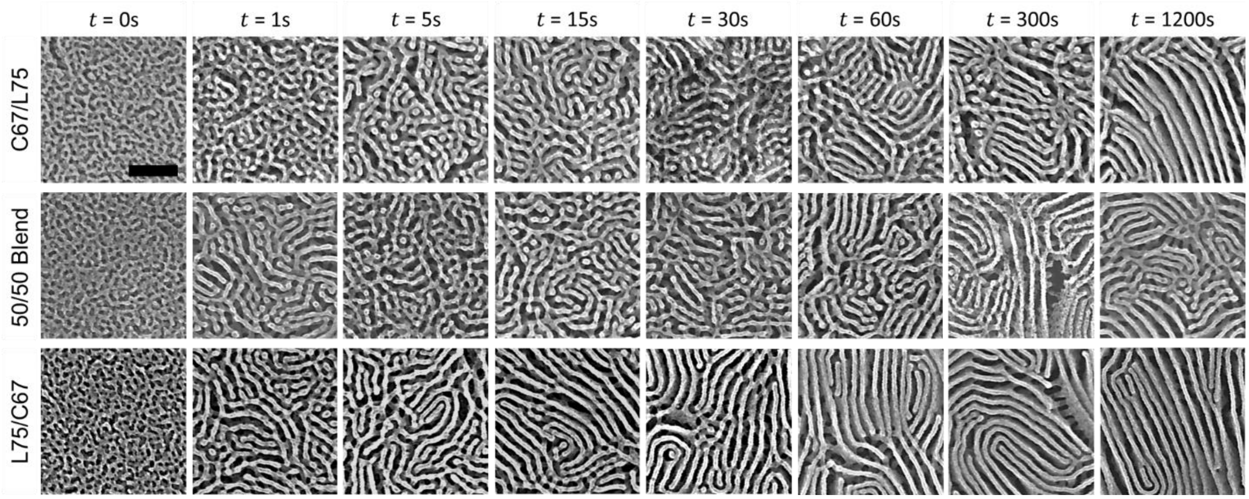

Figure 33| Top-down SEM images AZO SIS replicas made from L75/C67 and C67/L75 bilayers and 1:1 blend of L75:C67 films annealed at $\mathrm{T} = 250$ ℃. Each column represents a different annealing time (t) and provides a representative structure for each sample at that time point. The devices were made on top of these samples by patterning metal electrodes using standard lift-off technique and the electrical conductivity of these structures were measured. The scale bar is 200nm and applies to all images.

	C67/L75 Bilayer			50/50 Blend			L75/C67 bilayer		
Annealing time (t)	ϕ_{C67}	$h_{Bilayer}$ (nm)	h_{AZO} (nm)	ϕ_{C67}	$h_{Bilayer}$ (nm)	h_{AZO} (nm)	ϕ_{C67}	$h_{Bilayer}$ (nm)	h_{AZO} (nm)
0s	0.53	153	150	0.50	150	146	0.52	145	144
1s	0.53	153	120	0.50	150	116	0.52	145	122
5s	0.52	149	118	0.50	148	113	0.51	148	125
15s	0.52	149	118	0.50	148	117	0.51	148	123
30s	0.51	144	113	0.50	147	118	0.50	147	126
60s	0.51	144	119	0.50	147	115	0.50	147	123
300s	0.49	141	118	0.50	149	118	0.50	149	127
1200s	0.49	141	117	0.50	149	121	0.50	149	122

Table 3| The film physical properties corresponding to all images shown in Figure S33. $h_{Bilayer}$ and h_{AZO} are measurements of the film thickness after coating and after SIS/rapid thermal processing respectively.

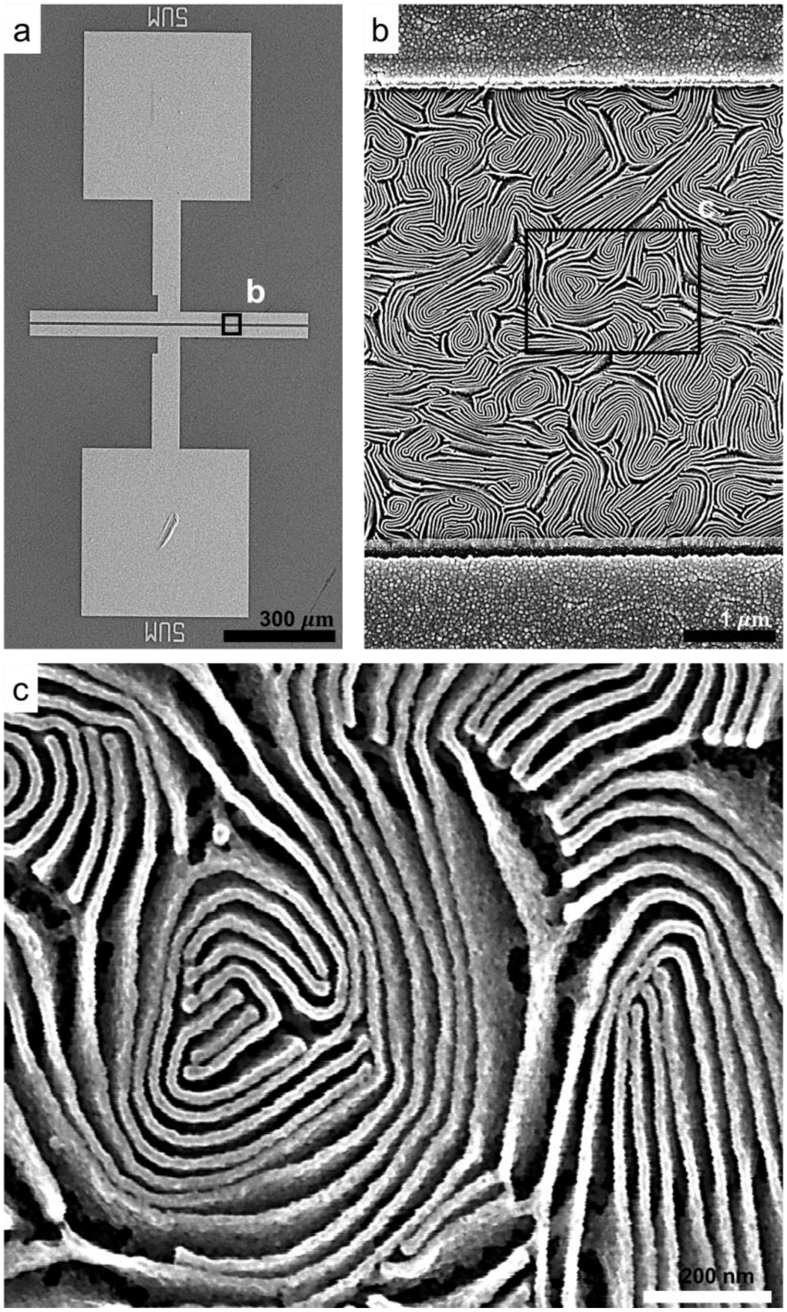

Figure 34| Top-down SEM images of L75/C67 bilayer film annealed for 1200s at $T = 250\ ^\circ C$ showing (a) an example image of the devices after fabrication (channel lengths varied from 500nm – 5 μm), (b) the BCP nanostructure in between the channel where electrical conduction is measured, and (c) and close-up image of the BCP nanostructure.

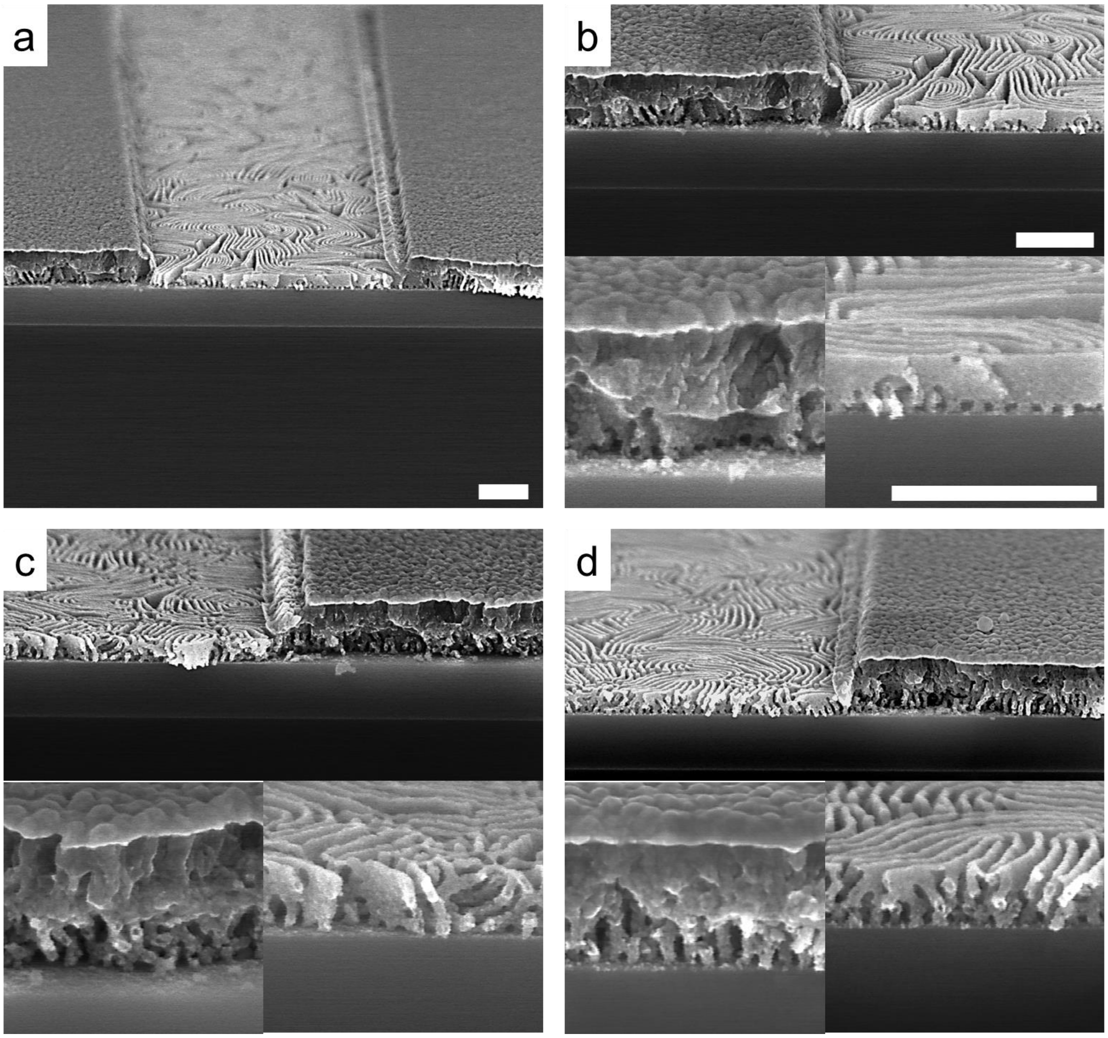

Figure 35| A series of SEM cross-sections examining the deposition of the electrical contacts onto the AZO film replicas. Note that silicon substrates with ~300 nm thermal oxide were used for electrical characterization and the oxide layer can be clearly seen in the cross-sectional SEM images. (a) an example image of the channel in which the conductivity measurements are performed. In this image we are examining electrical contacts deposited onto L75/C67 bilayer annealed for 1200s at $\mathrm{T} = 250\ ^{\circ}\mathrm{C}$. (b) SEM cross-section of L75/C67 bilayer annealed for 1200s at $\mathrm{T} = 250\ ^{\circ}\mathrm{C}$ of: (top) the AZO replica nanostructure in the conduction channel and underneath the electrical device, (bottom right) close-up of the conduction channel, and (bottom left) close-up of the structure underneath the electrical devices. The same imaging process was applied to (c) 1:1 blend of C67:L75 annealed for 1200s at $\mathrm{T} = 250\ ^{\circ}\mathrm{C}$ and (d) C67/L75 bilayer annealed for 1200s at $\mathrm{T} = 250\ ^{\circ}\mathrm{C}$. The scale bars in part b also apply to c and d and all scale bars are 400nm.

Supplementary References

1. Subramanian, A. *et al.* Three-dimensional electroactive ZnO nanomesh directly derived from hierarchically self-assembled block copolymer thin films. *Nanoscale* **11**, 9533–9546 (2019).
